# Blockchain adoption in remanufacturing under carbon tax policies: A game theory analysis of industry dynamics and consumer impact

**DOI:** 10.1371/journal.pone.0338919

**Published:** 2026-01-16

**Authors:** Jing Li, Tianchen Yang, Lihua Shi

**Affiliations:** 1 Shanghai Ocean University, Shanghai, China; 2 Shanghai University of Engineering Science, Shanghai, China; Yonsei University, KOREA, REPUBLIC OF

## Abstract

Within the context of China’s “dual carbon” goals, remanufacturing of waste products has garnered significant government interest due to its potential to reduce carbon emissions during production and alleviate environmental pollution. This study analyzed the impact of carbon tax policies (carbon tax and carbon tax reduction) and consumer demand on the blockchain introduction strategies of manufacturers and remanufacturers by constructing a two-party evolutionary game model of “manufacturers-remanufacturers”. The key findings are as follows: (1) Under carbon tax policies, manufacturers and remanufacturers in different industries exhibit divergent strategies regarding blockchain adoption. Specifically, manufacturers in industries lacking carbon emission advantages are more inclined to introduce blockchain technology. Conversely, remanufacturers in industries possessing carbon emission advantages demonstrate a stronger preference for adopting blockchain technology. (2) Government strategies involving tax reductions and exemptions can effectively incentivize manufacturers and remanufacturers to adopt blockchain, thereby boosting the sales of remanufactured products and the recycling of waste products. (3) Consumers’ environmental awareness and acceptance of remanufactured products significantly influence manufacturers’ and remanufacturers’ blockchain adoption decisions, although the nature of this impact varies across industries. Based on these insights, this paper proposes targeted strategies to facilitate the low-carbon development of the manufacturing industry. Different from previous studies, this study considers the external factors of “blockchain applications” to further enrich the research on remanufacturing decisions considering different external factors under carbon tax policies. Based on the application of blockchain technology, the impact of carbon tax policies on remanufacturing decisions in different industries is further compared and analyzed.

## Introduction

In the context of “dual carbon” (China’s national goals to peak CO₂ emissions by 2030 and achieve carbon neutrality by 2060), promoting an environmentally friendly economy and reducing resource waste and pollution emissions is not only a positive response to the current environmental crisis, but also a key path to achieving sustainable development goals [1]. Over the past decade, the annual average growth rate of carbon emissions in China’s manufacturing industry has been 3.19% [2]. Remanufacturing—the industrial process of restoring end-of-life products to “like-new” condition, reduces production costs by 50%, energy consumption by 60%, raw material demand by 70%, and CO₂ emissions by 80% [3]. The “Opinions on Accelerating the Construction of a Waste Recycling System” issued by the General Office of the State Council of China in 2024 also emphasize the importance of waste recycling and propose multiple specific measures to promote the remanufacturing of waste equipment and trade in of old equipment. Therefore, the remanufacturing of waste products as a low-carbon, environmentally friendly, and resource-saving industrial development model for manufacturers has received increasing attention [4]. However, there are few existing studies exploring the impact of carbon tax policies on remanufacturing decisions from the perspective of blockchain applications. In addition, under carbon tax policies, different industries exhibit industry heterogeneity in carbon tax costs and response strategies to blockchain technology due to differences in carbon emissions in production and remanufacturing processes. Therefore, it is necessary to explore the impact of carbon tax policies on the decisions of manufacturers and remanufacturers in different industries from a blockchain perspective.

Under the promotion of government measures such as “Trade-in”, more and more original equipment manufacturers are participating in remanufacturing [5], such as Xerox, Canon, IBM, and HP [6]. Some manufacturers are limited by the production technology required for remanufacturing and choose to cooperate with remanufacturers, using authorized remanufacturing and outsourced remanufacturing models [7]. Blockchain technology, a decentralized, immutable ledger enabling transparent, tamper-proof record-keeping, enhances traceability of remanufactured products. With the popularization of remanufacturing, blockchain technology can make it more convenient for consumers to access and verify the material source, production process, quality inspection and other information of products in the remanufacturing industry, better understand the background and quality of products, enhance consumers’ trust in products, and thus increase their willingness to purchase remanufactured products [8]. For example, JD’s second-hand trading platform Love Recycling utilizes blockchain technology to provide quality inspection records for second-hand mobile phones, tablets, and other products, thereby reducing consumers’ concerns about product quality [9]. In addition, by utilizing blockchain technology, remanufacturers can efficiently recycle waste products, thereby reducing their costs in the recycling and remanufacturing process [10].

To curb the increase in emissions, many countries have implemented emission regulations and policies, such as carbon taxes and carbon total control and trading. Among these, the carbon tax, a policy tool that levies fees on carbon emissions generated during production, thereby incentivizing low-carbon practices, it is widely recognized as one of the most effective market-based mechanisms. [11]. Currently, more than 20 countries, including Canada and the United States, have implemented a 2-carbon tax policy [12]. As the world’s second-largest economy, China is actively stimulating the national carbon trading market and implementing a series of incentive policies and measures to promote the development of the remanufacturing industry for enterprises. Therefore, whether and how to implement carbon tax policies is an urgent problem that needs to be solved. In view of the above considerations, this article will study the implementation of carbon tax policies and the following three questions:

1) How will manufacturers’ remanufacturing decisions be affected by the dual pressure of carbon tax policies and blockchain application costs?
2) Does the low recognition of remanufactured products by consumers have an incentive effect on the use of blockchain?
3) Can the carbon tax reduction strategy increase the promotion rate of blockchain?

**Fig 1:**
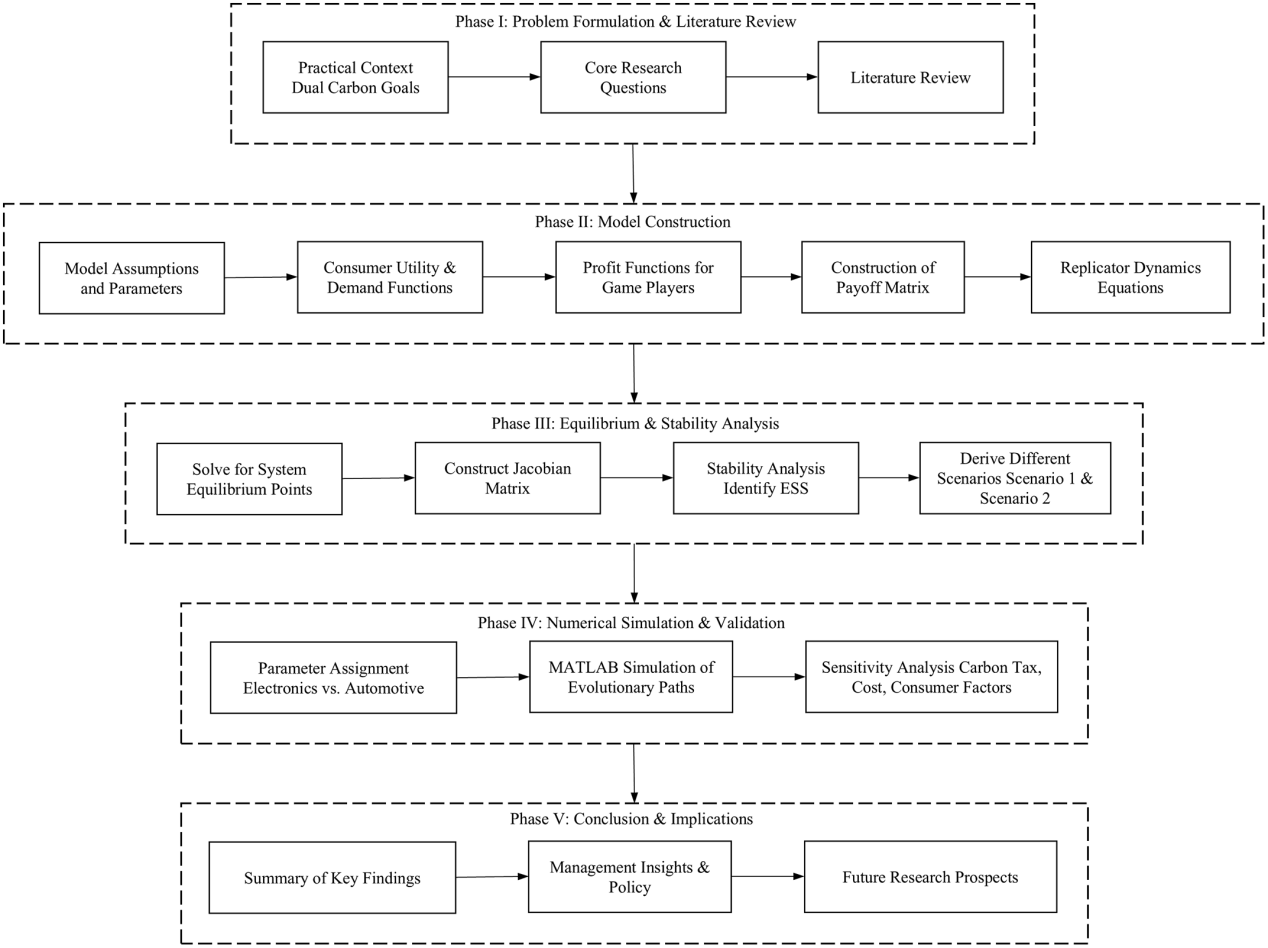
Flowchart.

The flowchart of this study is shown in Fig 1.

## Literature review

This study involves three aspects: remanufacturing decisions of manufacturers and remanufacturers under carbon taxes, the impact of blockchain technology on remanufacturing participants in supply chains, and the application of evolutionary game theory in remanufacturing supply chains.

### Carbon tax policy

Existing studies have conducted in-depth research on the remanufacturing decisions of manufacturers and remanufacturers under carbon taxes. Alegoz et al. focused on exploring production strategies and sustainability decision-making issues in pure manufacturing and hybrid manufacturing remanufacturing systems [13]. They also compared the overall system performance and the presentation of economic and environmental performance indicators by various parties in the supply chain in different contexts. In addition, scholars such as Li et al. also analyzed the impact of carbon tax policies on three different remanufacturing modes: independent remanufacturing, authorized remanufacturing, and outsourced remanufacturing [14]. They believe that under different carbon tax rates, choosing the appropriate remanufacturing mode can result in optimal benefits for manufacturers and remanufacturers. Sharma et al. conducted an in-depth analysis of the effects of carbon tax policies and remanufacturing strategies on reducing carbon emissions, and pointed out that adopting a decentralized model has more significant advantages in reducing carbon emissions compared to a centralized model [15]. In addition, they also believe that from a long-term perspective, implementing remanufacturing strategies is expected to bring profit growth opportunities for retailers and manufacturers. Duan et al. analyzed the impact of different carbon tax policies on participating companies in a closed-loop supply chain network by comparing the equilibrium conditions under two policies: a single progressive carbon tax and an excess progressive carbon tax [16]. Eslamipoor et al. analyzed the impact of a parallel mixed carbon policy of “carbon tax carbon trading” on remanufacturing decisions, and concluded that compared with existing carbon trading policies, the mixed carbon policy can further incentivize manufacturers to actively remanufacture and enhance the low-carbon reputation of products from a long-term perspective [17]. Kushwaha et al. considered the impact of patent authorization on remanufacturing decisions [18]. By comparing the operation of manufacturers and remanufacturers under three different policies of no carbon tax, implementation of carbon tax, and carbon quota and trading under patent authorization, they found that the carbon tax rate had the greatest impact on both parties’ decisions. Shekarian et al. considered the impact of retailers’ dual preferences on remanufacturing decisions under carbon tax policies, using manufacturer profits as a reference point, and analyzed the effects of changes in retailers’ loss aversion and negative unfairness psychology on upstream enterprise product wholesale prices [19]. Martin et al. considered the impact of consumer environmental awareness on outsourcing authorization remanufacturing decisions under carbon tax policies and believed that only when consumers have a high acceptance of remanufactured products, the price of remanufactured products will increase accordingly [20]. Tao et al. considered the impact of the trade in program on remanufacturing decisions under a carbon tax and believed that the trade in program could reduce the price sensitivity of environmentally conscious consumers [21]. They also determined the optimal carbon tax to be levied by regulatory agencies. To further enrich the remanufacturing decisions considering different external factors under the carbon tax policy, this study considers the external factor of “blockchain application” and further compares and analyzes the impact of carbon tax policy on remanufacturing decisions based on the application of blockchain technology.

### The application of blockchain technology

Regarding the application strategy of blockchain technology in the supply chain. Most research focuses on the analysis of the impact of blockchain technology on supply chain operations and finds that blockchain can improve supply chain performance [22], reduce supply chain operational risks [23], and mitigate moral hazard [24]. A small number of scholars have studied the application strategies of blockchain technology in the supply chain. Upadhyay et al. studied the blockchain adoption strategies of initial and new retailers and analyzed the impact of privacy breach risk on blockchain adoption [25]. Wu et al. studied the adoption strategies of blockchain technology and its impact on quality and pricing decisions in the competitive situation of strong and weak brand enterprises and found that whether two competing enterprises adopt blockchain technology depends most significantly on the adoption cost [26]. Qiao et al. found that when the cost of blockchain is relatively high or consumers are less sensitive to the benefits of blockchain or product recycling, companies will not invest in blockchain technology [27]. Biswas et al. found that whether the supply chain adopts blockchain technology is related to consumers’ traceability awareness, product production costs, and blockchain technology costs [28]. Different from the above research, this study will analyze the impact of carbon tax policies on the adoption strategy of blockchain in remanufacturing supply chains.

### The application of evolutionary game theory

Current scholars have conducted extensive research on remanufacturing supply chains and the application of blockchain technology using evolutionary game theory. Game theory, a mathematical framework for strategic interaction between rational agents [29]. This study constructs a two-party game model between manufacturers and remanufacturers to simulate the decision-making evolution process of both parties. In the field of remanufacturing supply chains, Li et al. constructed a two-echelon supply chain game model based on information asymmetry in the remanufacturing process, analyzing the game equilibrium strategies between manufacturers and retailers [30]. Wei et al. analyzed the game relationship between manufacturers and retailers, arguing that the equilibrium strategies between them are influenced by licensing fees and remanufactured product recovery efficiency [31]. Khan et al. examined the game relationship between original vehicle manufacturers and recyclers, concluding that the original manufacturers’ decision to license significantly impacts the equilibrium strategies of both parties [32]. Zhang et al. analyzed the game relationship among the government, waste battery remanufacturers, and consumers, suggesting that government subsidies for waste battery reuse can increase consumer benefits [33]. Huang et al. developed a two-party game model between manufacturers and consumers, simulating consumer recycling behavior and analyzing the impact of consumer recycling rates on manufacturer decisions [34]. Ma et al. established a two-party game model between remanufacturers and e commerce platforms, analyzing the influence of factors such as green innovation, recycling prices, and government subsidies on game equilibrium, concluding that stronger consumer price preference leads to higher recycling efficiency for remanufacturers [35]. Regarding the application of blockchain technology, Wang et al. constructed a two-party game model between suppliers and retailers, analyzing the impact of blockchain adoption costs and carbon quotas on the decisions of both parties [36]. Yang et al. used the electric vehicle industry as a case study to analyze the game relationship between suppliers and manufacturers, arguing that blockchain technology can promote green transformation in the supply chain [37]. Wei et al. based on blockchain data governance, analyzed a tripartite game relationship among the government, manufacturers, and retailers, finding that retailers’ sensitivity to blockchain adoption value has the most significant impact on the decisions of participants in the low carbon product supply chain [38]. Different from the above research, this study will analyze the game relationship between manufacturers and remanufacturers, and optimize the blockchain adoption strategy of the remanufacturing supply chain.

We summarize the most relevant literature in Table 1 and highlight the research gap. Although scholars have conducted extensive discussions on manufacturers’ remanufacturing from the perspective of carbon policies such as carbon taxes. However, few scholars have considered the impact of blockchain technology on the remanufacturing process under carbon tax policies, resulting in some limitations in the research on strategies to incentivize manufacturers and remanufacturers to introduce blockchain. Therefore, this article will construct an evolutionary game model between manufacturers and remanufacturers based on the introduction of blockchain and carbon tax policies. And using MATLAB software to simulate the game model, through system equilibrium analysis and sensitivity analysis, determine what equilibrium state each game subject ultimately reaches under the dual influence of blockchain technology application and carbon tax policy. Finally, based on the analysis results, a low-carbon development strategy is provided for the government to incentivize manufacturers and remanufacturers to introduce blockchain technology.

**Table 1:** Literature comparison.

Authors	Remanufacturing	Carbon Tax	Blockchain Technology	Evolutionary Game Theory
Eslamipoor et al. (2024) [17]	✓	✓		
Kushwaha et al. (2022) [18]	✓	✓		
Tao et al. (2024) [21]	✓	✓		
Qiao et al. (2023) [27]	✓		✓	
Biswas et al. (2022) [28]			✓	
Zhang et al. (2022) [33]	✓			✓
Huang et al. (2024) [34]	✓			✓
Ma et al. (2024) [35]	✓			✓
Wang et al. (2024) [36]			✓	✓
Yang et al. (2024) [37]			✓	✓
Wei et al. (2023) [38]			✓	✓
This Study	✓	✓	✓	✓

## Evolutionary game theory: Definition and its application

Evolutionary game theory, a blend of traditional game theory and evolutionary biology, replaces the “complete rationality” assumption with the more realistic “bounded rationality”. It holds that decision-making agents (e.g., enterprises) adjust strategies via dynamic learning, imitation, and trial-and-error to achieve group stability. Its core components include: participating groups (manufacturers and remanufacturers here), strategy sets (manufacturers: {adopt blockchain (MQ), not adopt (MN)}; remanufacturers: {adopt blockchain (RQ), not adopt (RN)}), and payoffs (profits under different strategies). These payoffs are shaped by two critical contextual factors: industry dynamics, which capture the evolving decisions of manufacturers and remanufacturers across different industries under carbon tax policies, and consumer impact, which reflects how consumer recognition (*δ*) of remanufactured products impacts strategic choices. The system’s evolution is formally described by replicator dynamics equations, which link the growth rate of a strategy to its relative payoff, while the ultimate resting points of this process—the evolutionary stable strategies (ESS)—are identified via Jacobian matrix eigenvalue analysis (Table 3). In this study, evolutionary game theory serves as the core theoretical framework, acting through four steps:

1. Model Construction: Based on bounded rationality (agents cannot find optimal solutions initially), a scenario involving the government, manufacturers, remanufacturers, and consumers is built. Mathematical modeling derives profits under four strategy combinations (NN, NQ, QN, QQ) to form a payoff matrix (Table 2), the basis for dynamic analysis.
2. Dynamic Evolution: Replicator dynamics equations (F(*x*)=d*x*/d*t*, F(*y*)=d*y*/d*t*) quantify how the proportions of manufacturers (*x*) and remanufacturers (*y*) adopting blockchain change over time.
3. Equilibrium Stability Analysis: Setting the equations to zero identifies five equilibrium points (E_1_-E_5_). Using the Jacobian matrix and Lyapunov theory, ESS (where all eigenvalue real parts are negative) is determined. The final stable state depends on key parameters (carbon tax t, blockchain cost *c*_*q*_, etc.), forming two scenarios.
4. Numerical Simulation: MATLAB-generated evolutionary path diagrams (Figs. 313) verify theoretical conclusions. Sensitivity analysis (adjusting *c*_*q*_, *t*, etc.) reveals external factors’ impact on decision-making, addressing core research questions and supporting management implications.

**Table 2:** Symbols and their meanings.

Symbol	Symbol meaning
t	The government levies carbon tax rates
γ	Carbon tax reduction ratio
f	Unit product patent authorization fee
$p_{n}^{i}$, $p_{r}^{i}$	Unit new/remanufactured product price
$q_{n}^{i}$, $q_{r}^{i}$	Production of new/remanufactured products
$c_{q}$	The cost of introducing blockchain technology
$c_{f}$	The cost of recycling waste products
$c_{n}$, $c_{r}$	Unit production cost of new/remanufactured products
$e_{n}$, $e_{r}$	Carbon emissions of new/remanufactured products by production units
δ	Consumer recognition of remanufactured products
α	The degree of information disclosure by remanufacturers regarding their products
g	Consumer sensitivity to product p $c_{q}$ roduction information
θ	Consumers’ environmental awareness
v	Consumer willingness to pay for the performance of new products
$\pi_{n}^{i}$, $\pi_{r}^{i}$	Manufacturer/remanufacturer revenue

## Model analysis

### Model assumptions

Under the carbon tax policy, consider a remanufacturing supply chain consisting of original manufacturers, consumers, and remanufacturers. Manufacturers, as leaders, are responsible for producing new products and determining the production volume and patent licensing fees based on the principle of maximizing profits. As followers, remanufacturers are responsible for recycling waste products and determining the production volume of remanufactured products after understanding the licensing fees and new product output. In addition, the prices of new and remanufactured products are determined by consumers’ willingness to purchase. Consumers, as demanders, choose to purchase new or remanufactured products based on the principle of maximizing utility.

In the authorization mode, remanufacturers need to pay an authorization fee of f to the original manufacturer to recycle and remanufacture waste products and sell remanufactured products. To further increase the sales volume of remanufactured products and reduce the cost of recycling and remanufacturing, remanufacturers can introduce blockchain technology. Before and after the introduction of blockchain technology, the impact of blockchain technology on the effectiveness of carbon tax policy implementation is determined by comparing the recycling rate of waste products and the revenue changes of manufacturers and remanufacturers based on carbon tax policies. Finally, consider introducing a carbon tax reduction strategy to implement carbon tax reductions for participants in the remanufacturing process who introduce blockchain technology. Under the carbon tax policy, both manufacturers and remanufacturers are required to pay carbon taxes to the government, and the government determines the tax amount based on the carbon emissions generated during the production process of both parties.

**Fig 2:**
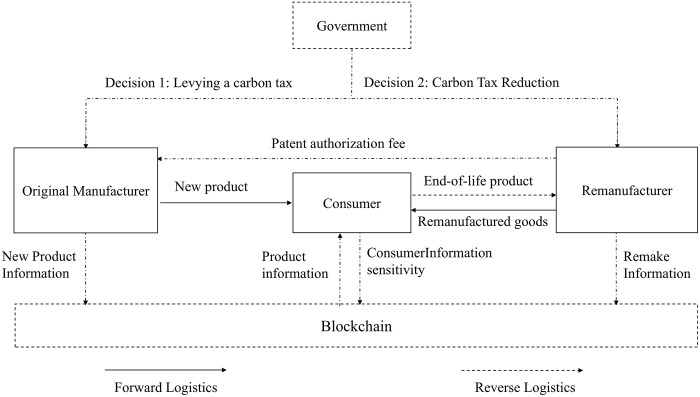
Game model of remanufacturing closed-loop supply chain considering the introduction of blockchain under carbon tax policy.

The game model of remanufacturing closed-loop supply chain is shown in Fig 2.

**Assumption 1.** The government levies a carbon tax based on the carbon emissions of products produced by manufacturers and remanufacturers, with a carbon tax rate of *t*. In addition, under the tax reduction strategy, the government provides carbon tax reductions to manufacturers or remanufacturers who introduce blockchain technology, with a carbon tax reduction rate of γ∈[0,1].

**Assumption 2.** There are two types of manufacturers: “introducing blockchain (MQ)” and “not introducing blockchain (MN)”, the x∈[0,1] probability of the manufacturer choosing “introducing blockchain” is, and the probability of choosing “not introducing blockchain” is 1−x. Among them, the average output of new products produced by the original manufacturer in each production cycle is $q_{n}$, the corresponding sales price of the product is $p_{n}$, the unit production cost is $c_{n}$, and the carbon emissions per unit of new product produced is $e_{n}$. Blockchain can reduce carbon k∈[0,1] emissions in the production process of new products by optimizing the management of carbon emission data, improving the transparency and efficiency of carbon trading, and promoting low-carbon supply chain management. Assuming the reduction ratio of carbon emissions is, the carbon emissions of the new product at this time are $(1-k)e_{n}$.

**Assumption 3.** There are two options for remanufacturer: “introducing blockchain (RQ)” and “not introducing blockchain (RN)”, the y∈[0,1] probability of remanufacturer choosing “introducing blockchain” is, and the probability of choosing “not introducing blockchain” is 1−y. Among them, remanufacturers produce remanufactured products by recycling the waste products generated in the previous cycle and paying patent licensing fees to the original manufacturer f. Assuming the output of remanufactured products produced by the remanufacturer is $q_{r}$ ($q_{r}<q_{n}$), the corresponding price of remanufactured products is $p_{r}$ ($p_{r}<p_{n}$), the unit production cost is $c_{r}$, and the carbon emissions per unit of new product produced are $e_{r}$ ($e_{r}<e_{n}$). After the introduction of blockchain by remanufacturers, the carbon emissions during the production process of remanufactured products have been reduced to $(1-k)e_{r}$. Referring to the research of Hrouga et al. the application of blockchain technology can reduce the unit production cost of remanufactured products [39]. Assuming that the introduction of blockchain reduces the unit production cost of remanufacturers producing remanufactured products to η∈(0,1).

**Assumption 4.** The willingness δ of consumers to pay for a new product *v* is, and since consumers generally believe that the quality of remanufactured products is inferior to that of new products, assuming that the consumer’s acceptance of the new product is 1, the acceptance of remanufactured products is δ∈(0,1), that is, the lower the acceptance, the lower the consumer’s willingness to pay. In addition, considering consumers’ awareness of environmental protection, their willingness to pay for products with high carbon emissions may decrease. Assuming that consumers’ awareness of environmental protection is θ∈[0,1]. Before the introduction of blockchain, remanufacturers used information disclosure about product production to enhance consumers’ recognition of the product. Assuming that consumers’ sensitivity to information about remanufacturing production is g∈[0,1],(δ+g<1), then before the introduction of blockchain, remanufacturers can increase consumers’ willingness to pay for remanufactured products from δv to (δ+gα)v by adopting information disclosure.

**Assumption 5.** The cost of introducing blockchain by the decision-making body is $c_{q}$. Before the introduction of blockchain, remanufacturers would disclose information about their products to improve product efficiency, thereby increasing consumers’ willingness to pay. Assuming that the degree of information disclosure by remanufacturers about their products is α∈[0,1]. After the introduction of blockchain, the degree of information disclosure α becomes 1.

**Assumption 6.** For industries with a carbon emission advantage, there is a significant disparity in the carbon emissions generated during the production of new products versus remanufactured products. Specifically, the carbon emissions from producing a new product per unit are substantially higher than those from producing a remanufactured product, indicating that a considerable amount of carbon emissions can be saved per unit of remanufactured product. Conversely, for industries without a carbon emission advantage, the carbon emissions generated during the production of new and remanufactured products are relatively similar, meaning that the carbon emissions saved per unit of remanufactured product are limited.

The symbols used and their meanings are shown in Table 2.

### Consumer utility function

Standardizing the market size to 1, considering the recognition of remanufactured products by demand side consumers, environmental awareness, sensitivity to production information disclosure, trust in production information disclosure, as well as the degree of information disclosure and the introduction of blockchain by supply side manufacturers and remanufacturers, it can be concluded that when manufacturers choose the “no blockchain” strategy (MN), the utility of consumers purchasing new products is:


$$V_{MN}=v-\theta e_{n}-p_{n}.$$ (1)

When manufacturers introduce blockchain (MQ), the utility of consumers purchasing new products is:


$$V_{MQ}=v-\theta (1-k)e_{n}-p_{n}.$$ (2)

Similarly, when remanufacturers choose the “no blockchain” strategy (RN), the utility of consumers purchasing remanufactured products is:


$$V_{RN}=(\delta +g\alpha )v-\theta e_{r}-p_{r}.$$ (3)

After the introduction of blockchain technology (RQ) by remanufacturers, the utility of consumers purchasing remanufactured products is:


$$V_{RQ}=(\delta +g)v-\theta (1-k)e_{r}-p_{r}.$$ (4)

Under the strategy combination (NN) $v_{1}<v<1$ of manufacturer and remanufacturer {without introducing blockchain, without introducing blockchain}, when $V_{MN}$ > $V_{RN}$ and $V_{MN}>0$, it can be obtained that $v>\frac{\theta (e_{n}-e_{r})+p_{n}-p_{r}}{1-(\delta +g\alpha )}$, and $v>p_{n}+\theta e_{n}$, let $v_{1}=\frac{\theta (e_{n}-e_{r})+p_{n}-p_{r}}{1-(\delta +g\alpha )}$, at that time, the consumer demand for the new product is:


$$q_{{}_{n}}^{NN}=\int_{v_{1}}^{1}f(v)dv=1-\frac{\theta (e_{n}-e_{r})+p_{n}-p_{r}}{1-(\delta +g\alpha )}.$$ (5)

$v_{2}<v<v_{1}$, $v_{2}=\frac{p_{r}+\theta e_{r}}{\delta +g\alpha }$, When $V_{MN}<V_{RN}$ and $V_{RN}>0$, the demand for remanufactured products from consumers is:


$$q_{{}_{r}}^{NN}=\int_{v_{2}}^{v_{1}}f(v)dv=\frac{\theta [(\delta +g\alpha )e_{n}-e_{r}]+(\delta +g\alpha )p_{n}-p_{r}}{\left(\delta +g\alpha )(1-\delta -g\alpha \right)}.$$ (6)

Similarly, the corresponding consumer demand for new and remanufactured products can be calculated for three strategy combinations: manufacturer and remanufacturer {without introducing blockchain, introducing blockchain} (NQ), {introducing blockchain, without introducing blockchain} (QN), and {introducing blockchain, introducing blockchain} (QQ)


$$q_{{}_{n}}^{NQ}=1-\frac{\theta [e_{n}-(1-k)e_{r}]+p_{n}-p_{r}}{1-(\delta +g)},$$

$$q_{{}_{r}}^{NQ}=\frac{\theta [(\delta +g)e_{n}-(1-k)e_{r}]+(\delta +g)p_{n}-p_{r}}{\left(\delta +g)(1-\delta -g\right)},$$

$$q_{{}_{n}}^{QN}=1-\frac{\theta [(1-k)e_{n}-e_{r}]+p_{n}-p_{r}}{1-(\delta +g\alpha )},$$

$$q_{{}_{r}}^{QN}=\frac{\theta [(\delta +g\alpha )(1-k)e_{n}-e_{r}]+(\delta +g\alpha )p_{n}-p_{r}}{\left(\delta +g\alpha )(1-\delta -g\alpha \right)},$$

$$q_{{}_{n}}^{QQ}=1-\frac{\theta (1-k)[e_{n}-e_{r}]+p_{n}-p_{r}}{1-(\delta +g\alpha )},$$

$$q_{{}_{r}}^{QQ}=\frac{\theta (1-k)[(\delta +g)e_{n}-e_{r}]+(\delta +g)p_{n}-p_{r}}{\left(\delta +g)(1-\delta -g\right)}.$$

Let $a_{1}=(\delta +g\alpha )$, $a_{2}=(\delta +g)$, $b_{1}=\theta (e_{n}-e_{r})$, $b_{2}=\theta (a_{1}e_{n}-e_{r})$, $b_{3}=\theta [e_{n}-(1-k)e_{r}]$, $b_{4}=\theta [a_{2}e_{n}-(1-k)e_{r}]$, $b_{5}=\theta [(1-k)e_{n}-e_{r}]$, $b_{6}=\theta [a_{1}e_{n}-e_{r}]$, $b_{7}=\theta (1-k)(e_{n}-e_{r})$, $b_{8}=\theta (1-k)(a_{2}e_{n}-e_{r})$.

### Profit functions of manufacturers and remanufacturers

In this section, both manufacturers and remanufacturers make decisions based on the principle of maximizing profits. Therefore, when the government adopts different carbon tax implementation strategies, the corresponding profit functions for manufacturers and remanufacturers are as follows:

In the policy combination (NN) of levying carbon taxes, not introducing blockchain, and not introducing blockchain among the government, manufacturers, and remanufacturers, the corresponding profit functions for manufacturers and remanufacturers are as follows:


$$\pi_{n}^{NN}=(p_{n}^{NN}-e_{n}t-c_{n})q_{{}_{n}}^{NN}+fq_{{}_{r}}^{NN},$$ (7)

$$\pi_{r}^{NN}=(p_{r}^{NN}-e_{r}t-c_{r}-f)q_{{}_{r}}^{NN}.$$ (8)

Since $\frac{\partial^{2}\pi_{r}^{NN}}{\partial p_{r}^{NN^{2}}}=\frac{2(\delta +g\alpha -1)}{\delta }<0$, $\frac{\partial^{2}\pi_{n}^{NN}}{\partial p_{n}^{NN^{2}}}=\frac{\text{2}}{\delta +g\alpha -1}<0$, it is known that $\pi_{n}^{NN}$ and $\pi_{r}^{NN}$ are $p_{r}^{NN}$ convex functions of $p_{n}^{NN}$ and respectively. Therefore $\frac{\partial \pi_{r}^{NN}}{\partial p_{r}^{NN^{2}}}=0$, $\frac{\partial \pi_{n}^{NN}}{\partial p_{n}^{NN^{2}}}=0$, the simultaneous solution yields $p_{r}^{NN}$.

To simplify the calculation, let $c_{1}=2c_{n}+c_{r}$, $c_{2}=a_{1}c_{n}+2c_{r}$, $d_{1}=b_{2}-2b_{1}$, $d_{2}=2b_{2}-a_{1}b_{1}$, $T_{1}=(2e_{n}+e_{r})t$, $T_{2}=(a_{1}e_{n}+2e_{r})t$, $L_{1}=2f+a_{1}(1-a_{1})+c_{2}$, $H_{1}=f+2(1-a_{1})+c_{1}$, $S_{1}=-c_{\text{1}}+c_{2}+f+2(1-a_{1})$, $K_{1}=-c_{2}+a_{1}c_{1}-f+a_{1}(1-a_{1})$.


$$p_{{}_{n}}^{NN}=\frac{b_{2}-2b_{1}+2c_{n}+c_{r}+(2e_{n}+e_{r})t+f+2(1-a_{1})}{4-a_{1}}=\frac{d_{1}+T_{1}+H_{1}}{4-a_{1}},$$

$$p_{{}_{r}}^{NN}=\frac{2b_{2}-a_{1}b_{1}+a_{1}c_{n}+2c_{r}+(a_{1}e_{n}+2e_{r})t+2f+a_{1}(1-a_{1})}{4-a_{1}}=\frac{d_{2}+T_{2}+L_{1}}{4-a_{1}}.$$

Substituting into equations (5), (6), (7), and (8) yields:


$$q_{{}_{n}}^{NN}=\frac{T_{2}-T_{1}+d_{1}+S_{1}}{\left(1-a_{1})(4-a_{1}\right)}=\frac{b_{2}-2b_{1}+(a_{1}-2)c_{n}+c_{r}+[(a_{1}-2)e_{n}+e_{r}]t+f+2(1-a_{1})}{\left(1-a_{1})(4-a_{1}\right)},$$

$q_{{}_{r}}^{NN}=\frac{\left(1-a_{1})(a_{1}T_{1}-T_{2}+d_{2}+K_{1}\right)}{a_{1}(4-a_{1})}=\frac{\left(1-a_{1})(2b_{2}-a_{1}b_{1}+a_{1}c_{n}+a_{1}c_{r}-2c_{r}+a_{1}e_{n}t+(a_{1}-2)e_{r}t-f+a_{1}(1-a_{1})\right)}{a_{1}(4-a_{1})}.$ Substituting $q_{{}_{n}}^{NN}$ and $p_{{}_{r}}^{NN}$, into equations (7) and (8) yields $\pi_{n}^{NN}$ and $\pi_{r}^{NN}$.

Similarly, under the strategy combination (NQ) of government, manufacturer, and remanufacturer {not introducing blockchain, introducing blockchain}, the corresponding profit functions are as follows $K_{2}=-c_{3}+a_{2}c_{1}-f+a_{2}(1-a_{2})$:

$p_{{}_{n}}^{NQ}=\frac{d_{3}+T_{3}+H_{2}}{4-a_{2}},$ $p_{{}_{r}}^{NQ}=\frac{d_{4}+T_{4}+L_{2}}{4-a_{2}},$ $q_{{}_{n}}^{NQ}=\frac{T_{4}-T_{3}+d_{3}+S_{2}}{\left(1-a_{2})(4-a_{2}\right)},$ $q_{{}_{r}}^{NQ}=\frac{\left(1-a_{2})(a_{2}T_{3}-T_{4}+d_{4}+K_{2}\right)}{a_{2}(4-a_{2})},$


$$\pi_{n}^{NQ}=(p_{{}_{n}}^{TNQ}-e_{n}t-c_{n})q_{{}_{n}}^{NQ}+fq_{{}_{r}}^{NQ},$$

$$\pi_{r}^{NQ}=(p_{{}_{r}}^{NQ}-e_{r}t(1-k)(1-\gamma )-c_{r}-f)q_{{}_{r}}^{NQ}-c_{q}.$$

The corresponding profit functions for manufacturers and remanufacturers under the QN strategy combination of introducing blockchain and not introducing blockchain are as follows $T_{6}=[a_{1}e_{n}(1-k)(1-\gamma )+2e_{r}]t$:

$p_{{}_{n}}^{QN}=\frac{d_{5}+T_{5}+H_{1}}{4-a_{1}},$ $p_{{}_{r}}^{QN}=\frac{d_{6}+T_{6}+L_{1}}{4-a_{1}},$ $q_{{}_{n}}^{QN}=\frac{T_{6}-T_{5}+d_{5}+S_{1}}{\left(1-a_{1})(4-a_{1}\right)},$ $q_{{}_{r}}^{QN}=\frac{\left(1-a_{1})(a_{1}T_{\text{5}}-T_{\text{6}}+d_{6}+K_{1}\right)}{a_{1}(4-a_{1})},$


$$\pi_{n}^{QN}=(p_{n}-e_{n}t(1-k)-c_{n})q_{{}_{n}}^{QN}+fq_{{}_{r}}^{QN}-c_{q},$$

$$\pi_{r}^{QN}=(p_{r}-e_{r}t-c_{r}-f)q_{{}_{r}}^{QN}.$$

The corresponding profit functions under the strategy combination of manufacturer and remanufacturer {introducing blockchain, introducing blockchain} (QQ), $T_{8}=(a_{2}e_{n}+2e_{r})(1-k)(1-\gamma )t$ are as follows:

$p_{{}_{n}}^{QQ}=\frac{d_{7}+T_{7}+H_{2}}{4-a_{2}},$ $p_{{}_{r}}^{QQ}=\frac{d_{8}+T_{8}+L_{2}}{4-a_{2}},$ $q_{{}_{n}}^{QQ}=\frac{T_{8}-T_{7}+d_{7}+S_{2}}{\left(1-a_{2})(4-a_{2}\right)},$ $q_{{}_{r}}^{QQ}=\frac{\left(1-a_{2})(a_{2}T_{7}-T_{8}+d_{8}+K_{2}\right)}{a_{2}(4-a_{2})},$


$$\pi_{n}^{QQ}=(p_{n}-e_{n}t(1-k)(1-\gamma )-c_{n})q_{{}_{n}}^{QQ}+fq_{{}_{r}}^{QQ}-c_{q},$$

$$\pi_{r}^{QQ}=(p_{r}-e_{r}t(1-k)(1-\gamma )-c_{r}-f)q_{{}_{r}}^{QQ}-c_{q}.$$

### Construction of profit matrix

According to the model assumptions, the game benefit matrix for the government, manufacturers, and remanufacturers is obtained as shown in Table 3.

**Table 3:** Profit Matrix of Quadruple Game.

	Manufacturer MQ, x	Manufacturer MN, 1−x
Remanufacturer RQ y	$(p_{{}_{n}}^{QQ}-e_{n}t(1-k)(1-\gamma )-c_{n})q_{{}_{n}}^{QQ}+fq_{{}_{r}}^{QQ}-c_{q}$ $(p_{{}_{r}}^{QQ}-e_{r}t(1-k)(1-\gamma )-c_{r}-f)q_{{}_{r}}^{QQ}-c_{q}$	$(p_{{}_{n}}^{NQ}-e_{n}t-c_{n})q_{{}_{n}}^{NQ}+fq_{{}_{r}}^{NQ}$ $(p_{{}_{r}}^{NQ}-e_{r}t(1-\gamma )(1-k)-c_{r}-f)q_{{}_{r}}^{NQ}-c_{q}$
Remanufacturer RN 1−y	$(p_{{}_{n}}^{QN}-e_{n}t(1-\gamma )(1-k)-c_{n})q_{{}_{n}}^{QN}+fq_{{}_{r}}^{QN}-c_{q}$ $(p_{r}-e_{r}t-c_{r}-f)q_{{}_{r}}^{QN}$	$(p_{{}_{n}}^{NN}-e_{n}t-c_{n})q_{{}_{n}}^{NN}+fq_{{}_{r}}^{NN}$ $(p_{{}_{r}}^{NN}-e_{r}t-c_{r}-f)q_{{}_{r}}^{NN}$

### Construction of expected return function

Assuming that the manufacturer’s expected revenue under the “introduction of blockchain” strategy is $U_{MQ}$; The expected return under the strategy of “not introducing blockchain” is $U_{MN}$; The comprehensive income of the manufacturer is $\overset{―}{U_{M}}$, based on the above assumptions and symbolic explanations, it can be concluded that


$$U_{MQ}=y\{[p_{{}_{n}}^{QQ}-e_{n}t(1-k)-c_{n}]q_{{}_{n}}^{QQ}+fq_{{}_{r}}^{QQ}\}+(1-y)\{[p_{{}_{n}}^{QN}-e_{n}t(1-k)-c_{n}]q_{{}_{n}}^{QN}+fq_{{}_{r}}^{QN}\}-c_{q},$$

$$U_{MN}=y[(p_{{}_{n}}^{NQ}-e_{n}t-c_{n})q_{{}_{n}}^{NQ}+fq_{{}_{r}}^{NQ}]+(1-y)[(p_{{}_{n}}^{NN}-e_{n}t-c_{n})q_{{}_{n}}^{NN}+fq_{{}_{r}}^{NN}],$$

$$\overset{―}{U_{M}}=xU_{MQ}+(1-x)U_{MN}.$$

Therefore, the manufacturer’s replicated dynamic equation can be obtained as


$$F(x)=\frac{dx}{dt}=x(1-x)(U_{MQ}-U_{MN})$$

Assuming the expected revenue under the remanufacturer’s “introduction of blockchain” strategy is $U_{RQ}$; The expected return under the strategy of “not introducing blockchain” is $U_{RN}$; The comprehensive income of the remanufacturer is $\overset{―}{U_{R}}$, based on the above assumptions and symbolic explanations, it can be concluded that



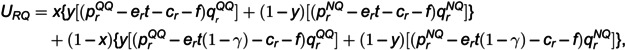





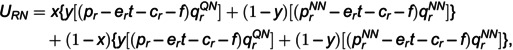




$$\overset{―}{U_{R}}=yU_{RQ}+(1-y)U_{RN}.$$

Therefore, the manufacturer’s replicated dynamic equation can be obtained as


$$F(y)=\frac{dy}{dt}=y(1-y)(U_{RQ}-U_{RN}).$$

## Analysis of evolutionary stability strategies

This article aims to explore the evolutionary stability strategies between equilibrium points in a two-party evolutionary game. Friedman et al. [29] pointed out that ESS only appears in pure strategies. Therefore, we studied the stability of five pure strategies in a two-party evolutionary game. The stability of equilibrium points in multiple dynamic systems can be obtained through Lyapunov stability theory. According to Lyapunov stability theory, the stability of the equilibrium point also needs to be obtained through local stability analysis of the Jacobian matrix. According to the replicator dynamics equations of various stakeholders, the Jacobian matrix *J* is obtained, which can be expressed as:

Among them, the values of each element in the Jacobian matrix are as follows.

Let F(x)=F(y)=0,obtain local equilibrium points E_1_ (0,0), E_2_ (1,0), E_3_ (0,1), E_4_ (1,1), E_5_ (*x*_*0*_, *y*_*0*_). According to evolutionary game theory, if the eigenvalues of the Jacobian matrix equation (17) are negative, then the equilibrium point is the stable point (ESS) of the evolutionary game. From this, Table 4 can be obtained.

**Table 4:** Eigenvalues of Jacobian Matrix.

Equilibrium point	Eigenvalue 1	Eigenvalue 2	Result
E_1_(0,0)	<0	>0	Unstable fixed point
E_2_(1,0)	<0	$<0(e_{n}=0.7,e_{r}=0.52)>0(e_{r}=5.2,e_{n}=0.52)$	ESS, Unstable fixed point
E_3_(0,1)	<0	$<0(e_{r}=5.2,e_{n}=0.52)>0(e_{n}=0.7,e_{r}=0.52)$	Unstable point, ESS
E_4_(1,1)	>0	>0	saddle point
E_5_(*x*_*0*_, *y*_*0*_)	>0	<0	Unstable fixed point

Under loose government regulations, the government only adopts corresponding policy measures for manufacturers, that is, the stability of each equilibrium point is mainly affected by carbon tax (*t*), at which point $0>\pi_{s}^{1}>\pi_{s}^{2}$, 0$>\pi_{r}^{1}>\pi_{r}^{2}$. This article analyzes the stability of the equilibrium point of the complex dynamic system based on the relationship between various parameters, as shown in Table 3.

**Scenario 1.** When $e_{r}=0.1,e_{n}=0.075$, it was found that the eigenvalues of the Jacobian matrix corresponding to equilibrium point E_2_ (1,0) were all non-positive values. Therefore, E_2_(1,0) was the stable point of system evolution, indicating that the blockchain adoption strategies of manufacturers and remanufacturers will remain stable when manufacturers adopt blockchain technology and remanufacturers do not adopt blockchain.

**Scenario 2.** When $e_{r}=5.2,e_{n}=0.52$, it was found that the eigenvalues of the Jacobian matrix corresponding to equilibrium point E_3_ (0,1) were all non-positive values. Therefore, E_3_ (0,1) is the stable point of system evolution, indicating that manufacturers and remanufacturers will remain stable when manufacturers do not adopt blockchain and remanufacturers adopt it.

The analysis of Scenario 1 and Scenario 2 reveals that manufacturers and remanufacturers do not adopt identical strategies, indicating that one party will inevitably take the lead in adopting blockchain technology.

## Numerical simulation analysis

To gain a more intuitive understanding of the evolutionary game process of each player, MATLAB software is used for simulation analysis. The prerequisites that need to be met during the model building process are $c_{r}<c_{n}$, $e_{r}<e_{n}$. For generality, the initial values of *x*, *y* are all set to 0.5. According to authoritative policy documents issued by the Central People’s Government of the People’s Republic of China and the latest data released by the Ministry of Ecology and Environment of China in 2021, and with reference to [13,40,41], the initial parameter settings are as follows: $c_{n}=0.5$, $c_{r}=0.2$, $c_{q}=0.2$, f=0.8; To maintain generality, this article takes electronic products and automotive engines as examples, and combines data from Asbahan et al. on e-readers, such as the fact that the energy consumption of newly manufactured e-readers is 1.33 times that of remanufactured electronic products [42]. After standardizing the data, carbon emission parameters are set for the electronic product remanufacturing industry: $e_{r}=0.1,e_{n}=0.075$; Yoshimura et al. found that in the process of remanufacturing automotive engines, the energy consumption of newly manufactured engines is 10 times that of remanufactured engines [43]. Based on this data, the carbon emission parameters for the automotive remanufacturing industry are set as $e_{r}=5.2,e_{n}=0.52$.

### Evolutionary path of stakeholders

To further analyze the role of carbon tax policies in promoting manufacturers and remanufacturers to adopt the “introduction of blockchain” strategy, the following simulation process will focus on the strategic changes of manufacturers and remanufacturers in different industries, aiming to clarify the key conditions that stakeholders need to meet to achieve evolutionary stable strategies. The parameter values for Scenario 1 and Scenario 2 are as follows.

**Fig 3:**
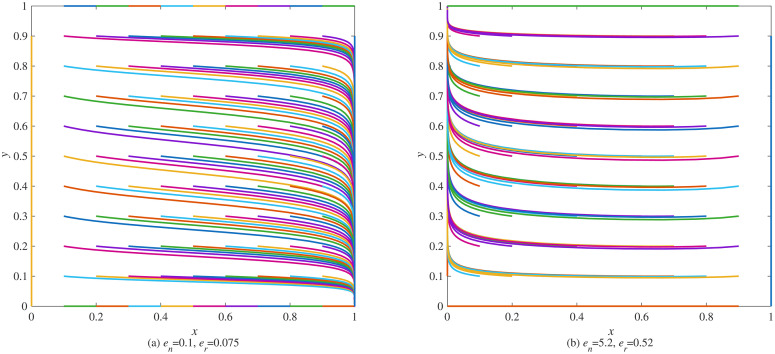
Evolution process of various strategies under strict government regulation. (a) *e*_*n*_ = 0.1, *e*_*r*_ = 0.075 (b) *e*_*n*_ = 5.2, *e*_*r*_ = 0.52.

**Scenario 1.** The system evolves to equilibrium E₂ (1,0), as shown in Fig 3 (a), where manufacturers adopt blockchain while remanufacturers do not. This occurs in industries with limited carbon emission advantages, such as electronics, where the carbon savings per remanufactured unit are relatively low. Here, manufacturers are more motivated to adopt blockchain to mitigate carbon tax pressures.

**Scenario 2.** The system stabilizes at equilibrium E₃ (0,1), as shown in Fig 3 (b), characterized by remanufacturers adopting blockchain while manufacturers abstain. This pattern emerges in industries with significant carbon emission advantages, such as automotive manufacturing, where remanufacturing yields substantial per-unit carbon savings. In such contexts, remanufacturers gain stronger incentives to leverage blockchain for enhancing operational transparency and reinforcing their environmental advantage.

### The impact of blockchain introduction cost (*c*_*q*_) on the remanufacturing process

**Fig 4:**
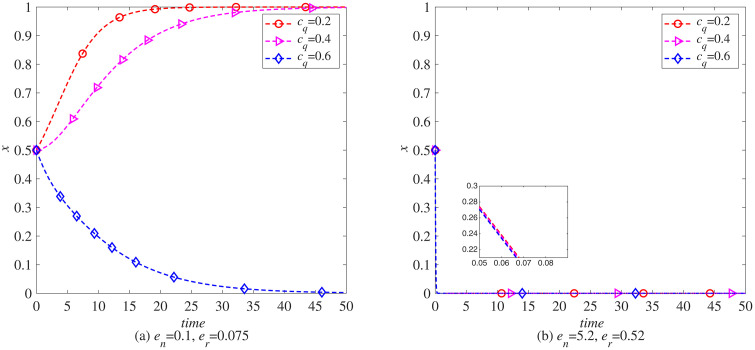
The evolution process of manufacturers introducing blockchain (x) under carbon tax policy. (a) *e*_*n*_ = 0.1, *e*_*r*_ = 0.075 (b) *e*_*n*_ = 5.2, *e*_*r*_ = 0.52.

**Fig 5:**
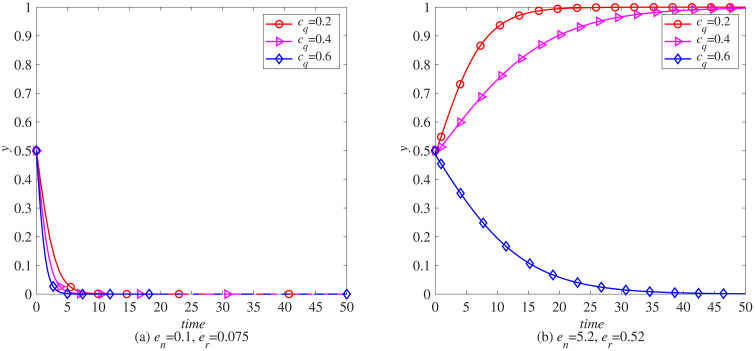
The evolutionary process of remanufacturers introducing blockchain (y) under carbon tax policy. (a) *e*_*n*_ = 0.1, *e*_*r*_ = 0.075 (b) *e*_*n*_ = 5.2, *e*_*r*_ = 0.52.

To analyze the impact of the cost of introducing blockchain on the remanufacturing process under carbon tax policies, this paper uses MATLAB software to conduct simulations. The parameters, t=0.5, γ=0, and the rest are set as initial values. That is, under the condition that the government does not implement carbon tax reduction, the evolution of the probabilities *x* and *y* of manufacturers and remanufacturers choosing to introduce blockchain is observed when the cost of introducing blockchain is $c_{q}=0.2,0.4,0.6$. As shown in Fig 4 (a), with the increase of the cost of introducing blockchain, the enthusiasm of manufacturers to choose to introduce blockchain gradually decreases. Based on the principle of maximizing profits, manufacturers in the electronic product industry start to give up introducing blockchain technology when the cost of introducing blockchain reaches 0.6. This also indicates that the cost of introducing blockchain will have a significant impact on whether manufacturers in the electronic product industry introduce blockchain technology. As shown in Fig 5 (a), with the increasing cost of introducing blockchain technology, the enthusiasm of remanufacturers to choose to introduce blockchain technology continues to decline, but the decline is not significant, indicating that the impact of blockchain introduction cost on whether remanufacturers in the electronic product industry introduce blockchain technology is not significant, that is, remanufacturers in industries without carbon emission advantages have stronger market adaptability.

In addition, the parameters *e*_*n*_ and *e*_*r*_ used in the above simulation process are determined with reference to the carbon emissions of newly manufactured and remanufactured products in the remanufacturing process of waste electronic products, that is, the energy consumption of newly manufactured electronic products is 1.33 times that of remanufactured electronic products. Unlike the electronics manufacturing industry, the carbon emissions of newly manufactured products in some industries are much higher than those of remanufactured products. Yoshimura et al. (2024) pointed out that in the process of remanufacturing automotive engines, the energy consumption of newly manufactured engines is 10 times that of remanufactured engines. Referring to this data, let parameter $e_{n}=5.2$, $e_{r}=0.52$, observe the impact of blockchain introduction costs on the automotive industry, as shown in Fig 4 (b). As the cost of blockchain introduction increases, manufacturers’ willingness to choose remanufacturing also gradually decreases. However, unlike electronic product manufacturing, automotive manufacturers are not sensitive to changes in blockchain introduction costs. This indicates that under the carbon tax policy, the remanufacturing process in the automotive industry is more adaptable to the market. As shown in Fig 5 (b), as the cost of blockchain increases, manufacturers’ enthusiasm for introducing blockchain also decreases. However, compared to the electronics industry, manufacturers in the automotive industry are more sensitive to changes in the cost of introducing blockchain.

#### Impact of carbon tax (*t*) on the remanufacturing process.

The implementation of carbon tax will increase the cost of carbon emissions in the remanufacturing process, requiring companies to consider energy consumption and carbon emissions more carefully when making remanufacturing decisions. This section explores in depth the impact of government carbon taxes on the remanufacturing decisions of manufacturers in different industries, using MATLAB software for simulation. Observe the impact of different carbon taxes (t=0, t=0.5, t=0.9) on the remanufacturing of the electronics and automotive industries separately, without the government implementing carbon tax reductions.

**Fig 6:**
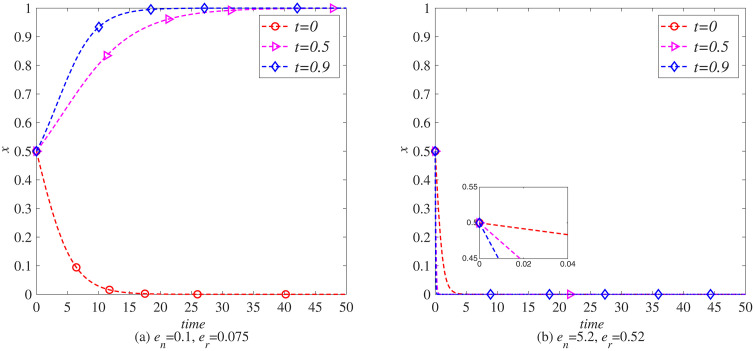
The evolution process of manufacturers introducing blockchain (x) under carbon tax policy. (a) *e*_*n*_ = 0.1, *e*_*r*_ = 0.075 (b) *e*_*n*_ = 5.2, *e*_*r*_ = 0.52.

**Fig 7:**
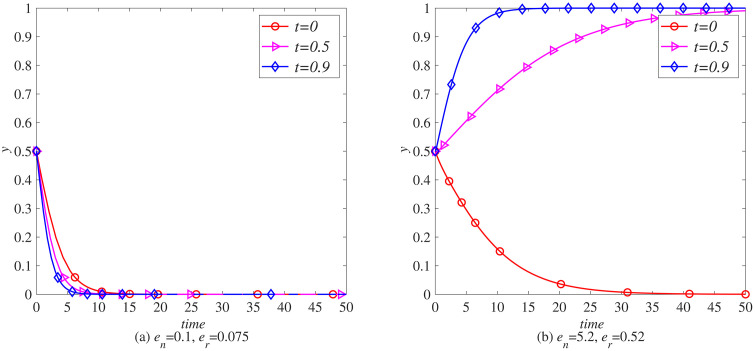
The evolutionary process of remanufacturers introducing blockchain (y) under carbon. (a) *e*_*n*_ = 0.1, *e*_*r*_ = 0.075 (b) *e*_*n*_ = 5.2, *e*_*r*_ = 0.52.

When $e_{n}=0.1$, $e_{r}=0.075$, as shown in Fig 6 (a), when the carbon tax rate exceeds 0.5, manufacturers in the electronics industry gradually tend to introduce blockchain technology. Due to the lack of significant carbon emission advantages of waste electronic and electrical products, relying solely on carbon taxes to incentivize manufacturers to introduce blockchain technology will limit the development of the electronic product market. As shown in Fig 7 (a), when the carbon tax rate exceeds 0.5, remanufacturers in the electronic product industry are gradually inclined to introduce blockchain. However, due to the low carbon emissions in the remanufacturing process of the electronic product industry, the impact of carbon tax on it is not significant, that is, the impact of carbon tax on remanufacturers in industries without obvious carbon emission advantages is not significant.

When $e_{n}=5.2$, $e_{r}=0.52$, in the in-depth analysis of Fig 6 (b), as the carbon tax level continues to rise, automobile manufacturers are facing a heavier carbon tax burden. Due to this cost pressure, although blockchain technology has potential advantages in improving efficiency and transparency, manufacturers have found that the economic benefits brought by its introduction are not as significant compared to the high carbon tax expenditures. Therefore, compared to the situation in Fig 6 (a), there has been a significant decrease in the sensitivity of automobile manufacturers to carbon tax policies, indicating that high carbon taxes have to some extent weakened manufacturers’ enthusiasm for technological innovation and change. However, when we turn to the scenario shown in Fig 7 (b), we discover a completely different trend. In the remanufacturing industry that already has carbon emission advantages, the increase of carbon tax has not become an obstacle, but rather a strong incentive factor. Remanufacturers in these industries actively respond to policy guidance, recognizing that the introduction of blockchain technology can further optimize resource allocation, improve operational efficiency, and effectively manage and reduce carbon emissions, thereby maintaining or enhancing their advantageous position in fierce market competition. Therefore, the increase in carbon tax has significantly incentivized remanufacturers in these industries to introduce blockchain technology, promoting innovation and application of green technology.

In summary, the carbon tax policy has had different impacts on the decisions of manufacturers and remanufacturers in the electronic and automotive industries to introduce blockchain technology. In the electronics industry, although the carbon emissions from waste products and remanufacturing processes are relatively low, carbon tax policies still encourage manufacturers and remanufacturers to introduce blockchain technology, but the impact is limited. In contrast, manufacturers in the automotive industry are more sensitive to carbon tax policies, and the high cost of carbon taxes has to some extent suppressed their enthusiasm for technological innovation and change. However, for the remanufacturing industry with low carbon emissions, the increase in carbon tax has become a powerful driving force for the introduction of blockchain technology, prompting remanufacturers in these industries to optimize resource allocation, improve operational efficiency, and effectively reduce carbon emissions through technological innovation, thereby gaining an advantage in market competition. This indicates that carbon tax policies play a complex and important role in guiding technological innovation and green development in different industries.

### The impact of carbon tax reduction (*γ*) on the remanufacturing process

To further explore the impact of the government’s implementation of carbon tax reduction measures on the remanufacturing process, this section uses MATLAB software for simulation. Taking the remanufacturing of waste electronic products with weak adaptability to supply chain interruption risks as an example, various parameters are set as initial values, and the impact of different carbon tax reduction ratios (γ=0, γ=0.5, γ=0.9) on the remanufacturing processes of the electronic product industry and the automotive industry is observed under the condition of levying a carbon tax rate of.

**Fig 8:**
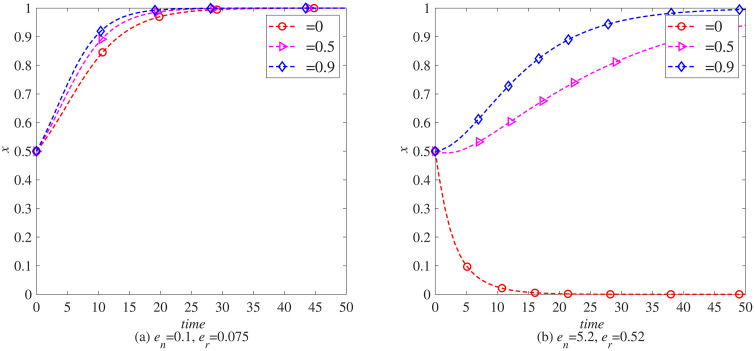
The evolution process of manufacturers introducing blockchain (x) under carbon tax policy. (a) *e*_*n*_ = 0.1, *e*_*r*_ = 0.075 (b) *e*_*n*_ = 5.2, *e*_*r*_ = 0.52.

**Fig 9:**
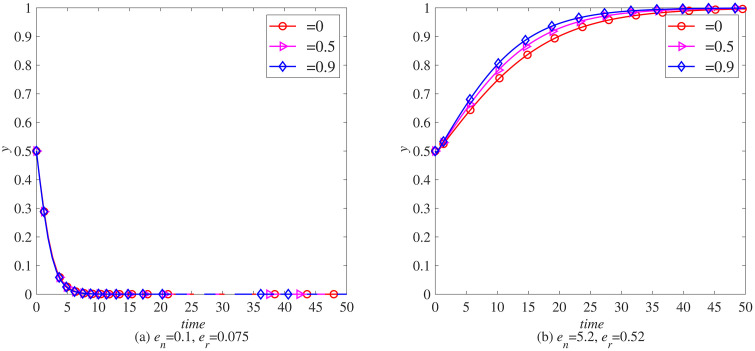
The evolutionary process of remanufacturers introducing blockchain (y) under carbon tax policy. (a) *e*_*n*_ = 0.1, *e*_*r*_ = 0.075 (b) *e*_*n*_ = 5.2, *e*_*r*_ = 0.52.

When $e_{n}=0.1$, $e_{r}=0.075$, as shown in Fig 8 (a) and Fig 9 (a), considering that the electronic product industry has not shown significant advantages in carbon emissions, manufacturers and remanufacturers generally maintain low sensitivity to adjustments to carbon tax policies. With the gradual increase in carbon tax reduction rates, although manufacturers and remanufacturers are more inclined to consider introducing blockchain technology to some extent, this change in inclination is quite subtle and has not had a significant impact on their strategic choices. The introduction of blockchain technology is more based on its inherent operational efficiency improvement, transparency enhancement, and potential long-term benefits considerations, rather than being directly driven by carbon tax reduction policies. Therefore, although the carbon tax reduction policy has brought certain positive incentives to the industry, manufacturers and remanufacturers in the electronic product industry still need to consider multiple factors comprehensively in the decision-making process to make the most appropriate strategic choices.

When $e_{n}=5.2$, $e_{r}=0.52$, as shown in Fig 9 (a) and Fig 9 (b), with the significant increase of carbon tax reduction ratio, the strategic choices of manufacturers and remanufacturers have undergone significant changes. This policy adjustment greatly enhances the willingness of both parties to introduce blockchain technology, making it their preferred path to optimize operations and enhance competitiveness. Blockchain technology can not only reduce operating costs by improving transparency and efficiency, but also play a key role in carbon footprint management, helping companies to further reduce carbon emissions and achieve sustainable development while enjoying carbon tax exemptions.

In summary, there are distinct differences in the attitudes of manufacturers and remanufacturers towards the introduction of blockchain technology in the electronic product industry under different carbon tax reduction policies. When the carbon tax reduction rate is low, although the policy brings certain positive incentives, the decisions of manufacturers and remanufacturers are more based on the operational advantages and long-term benefits of blockchain technology itself, and their sensitivity to carbon tax policies is relatively low. However, with the significant increase in carbon tax reduction rates, blockchain technology has become a key choice for manufacturers and remanufacturers to optimize strategies and enhance competitiveness due to its multiple advantages in reducing operating costs, enhancing transparency, and promoting sustainable development. Policy adjustments have significantly increased their willingness to introduce this technology, reflecting the synergistic effect of policy guidance and technological innovation in promoting green development in the industry.

### The impact of consumer willingness to pay (δ) on the remanufacturing process

To further explore the impact of consumer willingness to pay on the remanufacturing process under carbon tax policies, this section uses MATLAB software for simulation. Set each parameter as initial values and observe the impact of different consumer willingness to pay (δ=0.3, δ=0.4, δ=0.5) on the remanufacturing process under the government’s carbon tax rate of *t* = 0.5 and no carbon tax reduction.

**Fig 10:**
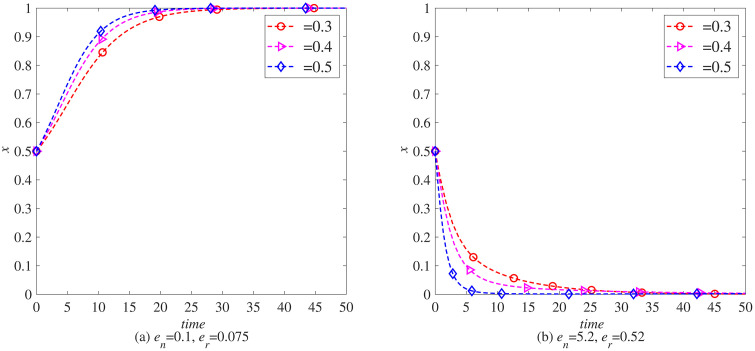
The evolution process of manufacturers introducing blockchain (x) under carbon tax policy. (a) *e*_*n*_ = 0.1, *e*_*r*_ = 0.075 (b) *e*_*n*_ = 5.2, *e*_*r*_ = 0.52.

**Fig 11:**
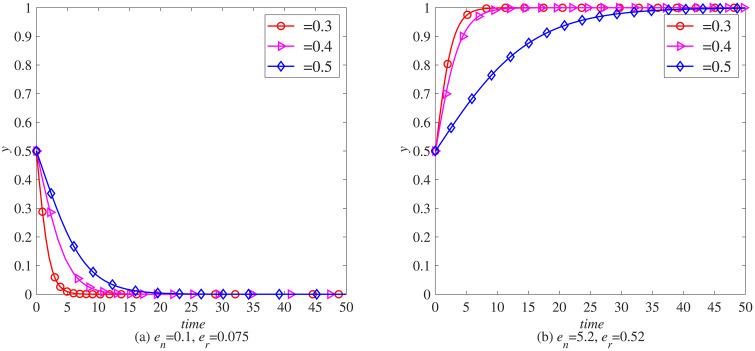
The evolutionary process of remanufacturers introducing blockchain (y) under carbon tax policy. (a) *e*_*n*_ = 0.1, *e*_*r*_ = 0.075 (b) *e*_*n*_ = 5.2, *e*_*r*_ = 0.52.

When $e_{n}=0.1$, $e_{r}=0.075$, as shown in Fig 10 (a) and Fig 11 (a), with the continuous increase in consumer willingness to pay, manufacturers and remanufacturers in the electronic product industry are also gradually increasing their willingness to introduce blockchain technology. This trend reflects the growing demand of consumers for high-quality, high transparency products, prompting manufacturers to actively explore the use of blockchain technology to improve the efficiency and credibility of product traceability, anti-counterfeiting verification, and supply chain management.

However, in sharp contrast, we can see in Fig 10 (b) and Fig 11 (b) that as consumers’ willingness to pay increases, manufacturers and remanufacturers in the automotive industry show a decreasing trend in their willingness to introduce blockchain technology. This phenomenon may be attributed to the cautious attitude of the automotive industry towards technological change, or the challenges and limitations of current blockchain technology applications in the automotive industry, such as high implementation costs, technical compatibility issues, and data privacy protection considerations, which have led manufacturers to not actively introduce blockchain technology in the face of increased consumer payment willingness, as seen in the electronics industry.

### The impact of consumer environmental awareness (θ) on the remanufacturing process

To further explore the impact of consumer environmental awareness on the remanufacturing process under carbon tax policies, this section uses MATLAB software for simulation. Set each parameter as initial values and observe the impact of different consumer environmental awareness (θ=0, θ=0.5, θ=0.9) on the remanufacturing process under the government’s carbon tax rate of *t* = 0.5 and no carbon tax reduction.

**Fig 12:**
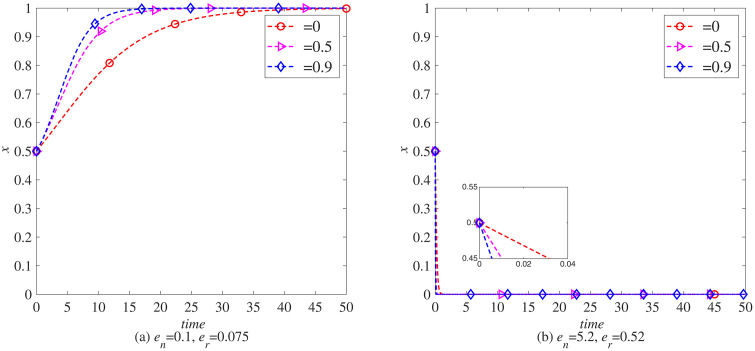
The evolution process of manufacturers introducing blockchain (*x*) under carbon tax policy. (a) *e*_*n*_ = 0.1, *e*_*r*_ = 0.075 (b) *e*_*n*_ = 5.2, *e*_*r*_ = 0.52.

**Fig 13:**
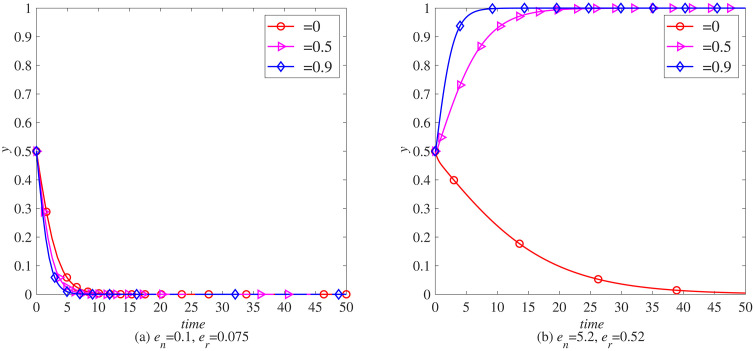
The evolutionary process of remanufacturers introducing blockchain (y) under carbon tax policy. (a) *e*_*n*_ = 0.1, *e*_*r*_ = 0.075 (b) *e*_*n*_ = 5.2, *e*_*r*_ = 0.52.

Through in-depth analysis of Figs 12 and 13, we can clearly see the positive incentive effect of consumer environmental awareness on manufacturers and remanufacturers in introducing blockchain technology. These two charts not only reveal the positive correlation between environmental awareness and technological innovation, but also further demonstrate the differentiated performance of this relationship in different industries.

Specifically, Figs 12 and 13 together indicate that as consumers increasingly prioritize environmental protection, they are becoming more inclined to support enterprises that adopt sustainable production methods and advanced technological means. This tendency is particularly evident in promoting manufacturers and remanufacturers to introduce blockchain technology, as blockchain technology, with its decentralization, high transparency, and strong traceability, provides more efficient and reliable solutions for product lifecycle management, helping enterprises achieve green production and circular economy.

Further comparing the data in Figs 12 (a) and 13 (a), we can find that in the electronic product industry, consumers’ environmental awareness has a particularly significant impact on manufacturers. This may be because electronic products are updated at a fast pace, and the pollution caused by discarded electronic products to the environment is becoming increasingly prominent. Therefore, consumers pay more attention to their environmental performance and corporate environmental responsibility when choosing electronic products. Manufacturers have to accelerate their pace of technological innovation in order to meet this market demand, and blockchain technology is one of the important means for them to enhance the environmental performance and competitiveness of their products.

However, when we turn our attention to the automotive industry, the situation is different. Comparing the data in Figs 12 (b) and 13 (b), it can be seen that consumers’ environmental awareness has a more significant impact on remanufacturers. This may be related to the particularity of the automotive industry. As a highly mature and competitive field, the automotive industry has relatively high technological barriers and costs for new car manufacturing, and remanufacturing has become a more economical and environmentally friendly production method. The increasing awareness of environmental protection among consumers makes them more inclined to choose car products that have been remanufactured, have good performance, and are reasonably priced. Therefore, remanufacturers face greater market demand and motivation in introducing blockchain technology to enhance product quality and traceability capabilities.

In summary, the increase in consumer environmental awareness not only motivates manufacturers and remanufacturers to introduce blockchain technology, but also presents differentiated impacts in different industries. This differentiated impact provides us with valuable insights: in the process of promoting green production and circular economy, enterprises should fully consider industry characteristics and market demand and introduce advanced technological means and innovative management models in a targeted manner to achieve a win-win situation of sustainable development and economic benefits.

## Conclusions and management insights

### Conclusion

Remanufacturing is an important pathway for low-carbon development in the manufacturing industry. This article considers the impact of consumer willingness to pay and the introduction of blockchain strategies on the remanufacturing process and constructs a two party evolutionary game model between manufacturers and remanufacturers under carbon tax policies. Using evolutionary game theory and MATLAB software to model and simulate the evolutionary process, this study analyzes the impact of factors such as carbon tax, carbon tax reduction, consumer willingness to pay, and consumer environmental awareness on the different strategic choices of manufacturers and remanufacturers under bounded rationality conditions. At the same time, several important conclusions were drawn and corresponding low-carbon development strategies were proposed based on them, aiming to incentivize manufacturers and remanufacturers to introduce blockchain technology and provide reference for the low-carbon and sustainable development of the manufacturing industry.

(1) Differences in the introduction strategies of blockchain technology under carbon tax policies. Under the framework of carbon tax policy, different industries face different cost pressures and market opportunities due to their carbon emission characteristics. For industries with traditionally high carbon emissions, such as steel and chemicals, manufacturers often face higher carbon tax burdens, which prompts them to actively seek ways to reduce carbon emissions. The introduction of blockchain technology can help manufacturers in these industries reduce their carbon footprint and thus alleviate their tax burden by optimizing supply chain management, improving production transparency and efficiency. Therefore, manufacturers in these industries are more inclined to introduce blockchain technology to achieve the dual goals of energy conservation, emission reduction, and cost control. On the contrary, in industries with carbon emission advantages such as clean energy and environmental technology, manufacturers themselves already have lower levels of carbon emissions and are less affected by carbon tax policies. However, remanufacturers in these industries may see opportunities to further improve product traceability and enhance consumer trust through blockchain technology, thereby promoting the sales of remanufactured products. The immutability of blockchain makes the source, ingredients, and quality information of remanufactured products more transparent, meeting consumers’ dual pursuit of environmental protection and quality.
(2) The incentive effect of government tax reduction and exemption strategies. To further encourage manufacturers and remanufacturers to adopt blockchain technology, the government can implement tax reduction strategies as incentive measures. This strategy can not only directly reduce the operating costs of enterprises, but also stimulate their innovation drive through policy guidance. Specifically, the government can provide tax exemptions or subsidies to enterprises that introduce blockchain technology and successfully implement energy conservation and emission reduction measures or increase their market share in remanufactured products. Such policies can not only promote the widespread application of blockchain technology in manufacturing and remanufacturing industries, but also accelerate the recycling and reuse of waste products, promoting the development of circular economy.
(3) The diverse impact of consumer environmental awareness and recognition. Consumers’ environmental awareness and recognition of recycled products are important factors affecting the introduction of blockchain technology by remanufacturers. With the deepening of environmental protection concepts, more and more consumers are paying attention to the environmental attributes and sustainability of products. Blockchain technology enhances consumers’ trust in remanufactured products and improves their market acceptance by providing transparent and trustworthy product traceability information. However, this impact varies across different industries. For example, in industries with high consumer attention such as fashion and electronics, the application of blockchain technology can better stimulate consumers’ willingness to purchase; In some traditional industrial sectors, consumers’ environmental awareness may not have reached a level that significantly influences their purchasing decisions.

### Management insights

(1) Manufacturers and remanufacturers should fully consider their own industry characteristics and carbon emission advantages, and reasonably formulate the introduction strategy of blockchain technology under the framework of carbon tax policy. For industries with low carbon emission savings, manufacturers should actively seek technological innovation and use blockchain to improve operational efficiency and emission reduction capabilities; For industries with high carbon emission savings, remanufacturers should fully utilize blockchain technology to optimize their remanufacturing processes and maintain or enhance their carbon emission advantages.
(2) The government should continue to improve its carbon tax policy and consider implementing incentive measures such as tax reductions to encourage manufacturers and remanufacturers to introduce low-carbon technologies such as blockchain. At the same time, the government should strengthen policy promotion, improve enterprises’ understanding and response capabilities to policies, and promote the implementation of policies.
(3) Enterprises should pay attention to the impact of consumer environmental awareness and recognition of recycled products and enhance consumer awareness and acceptance of recycled products through strengthening market education and marketing methods. This not only enhances the market competitiveness of enterprises, but also promotes consumer demand for low-carbon products, forming a virtuous cycle.
(4) Beyond the introduction cost of blockchain, implementing blockchain technology in remanufacturing supply chains faces challenges such as interoperability between different platforms, regulatory uncertainties, data standardization, and stakeholder resistance to new technologies; to proactively mitigate these barriers, enterprises and policymakers should assess interoperability requirements early to select solutions compatible with existing supply chain systems, collaborate on industry-wide data standards for seamless information exchange, develop regulatory frameworks with governments to address compliance ambiguities, implement phased technology rollouts to manage scalability risks and demonstrate tangible benefits, and engage stakeholders through tailored training to overcome resistance to technological change.

### Research prospects

While this study provides theoretical insights using evolutionary game theory and simulation, it acknowledges several limitations that point to fruitful avenues for future research. Firstly, the reliance on simulation modeling necessitates empirical validation. Future research could validate these findings using real-world industrial data, conducting case studies or large-scale surveys within specific manufacturing sectors (e.g., automotive electronics, industrial machinery remanufacturing) to assess the actual drivers and barriers to blockchain adoption under carbon pricing mechanisms. Secondly, the current model focuses solely on carbon tax policy. Extending the analysis to incorporate carbon trading markets represents a critical direction. Future work could develop comparative models or integrated frameworks examining how blockchain adoption incentives shift under cap-and-trade systems versus carbon taxes, and how blockchain might facilitate the verification and trading of carbon credits within remanufacturing supply chains. Finally, the model simplifies consumer behavior. Research incorporating richer behavioral factors is needed, such as empirical investigation into how blockchain verification actually influences consumer trust and willingness-to-pay across different product categories, or exploring the impact of information asymmetry and greenwashing concerns more deeply. Addressing these limitations will enhance the practical applicability and robustness of the findings.

## Supporting information

S1 File Numerical simulation code of Figs 3–12. (DOCX)
