# Supplementary material for: Blockchain adoption in remanufacturing under carbon tax policies: A game theory analysis of industry dynamics and consumer impact
Source: PLoS One. 2026 Jan 16;21(1):e0338919. doi: 10.1371/journal.pone.0338919 (PMC12810849; doi:10.1371/journal.pone.0338919)
Supplement: S1 File — (DOCX) [file pone.0338919.s001.docx]

Fig.3

clear; clc; % Clear workspace, clear command line

% Create figure and adjust layout

figure;

% Adjust subplot position (leave 20% space at the bottom)

subplot('Position', [0.1, 0.15, 0.35, 0.8]); % [left, bottom, width, height]

% Phase portrait of the first system (en=0.1, er=0.075)

for i=0:0.1:1 % Value range of x

for j=0:0.1:1 % Value range of y

[v,y] = ode45('qukuailian_R_s', [0 50], [i j]); % Solve the first equation

plot(y(:,1), y(:,2), 'linewidth', 1.5); % Plot trajectory

hold on; % Keep current figure

end

end

% Set properties of the first subplot

axis([0 1 0 1]);

xlabel('\it x', 'FontName', 'Times New Roman', 'FontSize', 10.5);

ylabel('\it y', 'FontName', 'Times New Roman', 'FontSize', 10.5);

set(gca, 'FontName', 'Times New Roman', 'FontSize', 10.5);

% Add title below the subplot (a)

text(0.5, -0.1, '(a) {\ite_n}=0.1, {\ite_r}=0.075', ... % Lower y-coordinate position

'FontName', 'Times New Roman', 'FontSize', 10.5, ...

'HorizontalAlignment', 'center', ...

'VerticalAlignment', 'top', ...

'Units', 'normalized');

% Second subplot (adjust position)

subplot('Position', [0.55, 0.15, 0.35, 0.8]); % Symmetric to the first subplot

% Phase portrait of the second system (en=0.52, er=0.052)

for i=0:0.1:1 % Value range of x

for j=0:0.1:1 % Value range of y

[v,y] = ode45('qukuailian_R', [0 50], [i j]); % Solve the second equation

plot(y(:,1), y(:,2), 'linewidth', 1.5); % Plot trajectory

hold on; % Keep current figure

end

end

% Set properties of the second subplot

axis([0 1 0 1]);

xlabel('\it x', 'FontName', 'Times New Roman', 'FontSize', 10.5);

ylabel('\it y', 'FontName', 'Times New Roman', 'FontSize', 10.5);

set(gca, 'FontName', 'Times New Roman', 'FontSize', 10.5);

% Add title below the subplot (b)

text(0.5, -0.1, '(b) {\ite_n}=5.2, {\ite_r}=0.52', ... % Lower y-coordinate position

'FontName', 'Times New Roman', 'FontSize', 10.5, ...

'HorizontalAlignment', 'center', ...

'VerticalAlignment', 'top', ...

'Units', 'normalized');

Fig.4

clear; clc; % Clear workspace, clear command line

% Create figure and adjust layout

figure;

% Adjust subplot position (leave 20% space at the bottom)

subplot('Position', [0.1, 0.15, 0.35, 0.8]); % [left, bottom, width, height]

% Phase portrait of the first system (en=0.1, er=0.075)

%%%%1

k=0.8;t=0.8;cn=0.5;cr=0.2;cq=0.2;g=0.4 ;a=0.5;er=0.52;en=0.7 ;derta=0.4;theta=0.5;f=0.8;gama=0.9;%Parameter assignment

[v,y]=ode45(@(v,y) liangfang_R_xiugai(v,y,k,t,cn,cr,cq,g,a,er,en,derta,theta,f,gama),[0,50],[0.5,0.5]); %Algorithm to solve the equation, equation running time (number of steps), initial values of x and y

p=plot(v,y(:,1),'ro--','linewidth',1.5,'markersize',8);

p.MarkerIndices = 1:10:length(y(:,1));%%%%Indicates the number of markers generated; the larger the middle value, the larger the marker interval

hold on

%%%%2

k=0.8;t=0.8;cn=0.5;cr=0.2;cq=0.35;g=0.4 ;a=0.5;er=0.52;en=0.7 ;derta=0.4;theta=0.5;f=0.8;gama=0.9;%Parameter assignment

[v,y]=ode45(@(v,y) liangfang_R_xiugai(v,y,k,t,cn,cr,cq,g,a,er,en,derta,theta,f,gama),[0,50],[0.5,0.5]); %Algorithm to solve the equation, equation running time (number of steps), initial values of x and y

p=plot(v,y(:,1),'m>--','linewidth',1.5,'markersize',8);

p.MarkerIndices = 1:10:length(y(:,1));%%%%Indicates the number of markers generated; the larger the middle value, the larger the marker interval

hold on

%%%%3

k=0.8;t=0.8;cn=0.5;cr=0.2;cq=0.6;g=0.4 ;a=0.5;er=0.52;en=0.7 ;derta=0.4;theta=0.5;f=0.8;gama=0.9;%Parameter assignment

[v,y]=ode45(@(v,y) liangfang_R_xiugai(v,y,k,t,cn,cr,cq,g,a,er,en,derta,theta,f,gama),[0,50],[0.5,0.5]); %Algorithm to solve the equation, equation running time (number of steps), initial values of x and y

p=plot(v,y(:,1),'bd--','linewidth',1.5,'markersize',8);

p.MarkerIndices = 1:10:length(y(:,1));%%%%Indicates the number of markers generated; the larger the middle value, the larger the marker interval

hold on

set(gca,'XTick',0:5:50,'YTick',0:0.1:1) %%[0:axis unit length:1]

axis([0 50 0 1])%%Range of horizontal and vertical axes

xlabel('time');%%Name of horizontal axis

ylabel('x');%%Name of vertical axis

legend('{\it c_{q}}=0.2','{\it c_{q}}=0.4','{\it c_{q}}=0.6');%Legend

% Set font properties for axes and labels

set(gca, 'FontName', 'Times New Roman', 'FontSize', 16);

xlabel('\it time', 'FontName', 'Times New Roman', 'FontSize', 16); % x-axis label

ylabel('\it x', 'FontName', 'Times New Roman', 'FontSize', 16); % y-axis label

% Add title below the subplot (a)

text(0.5, -0.12, '(a) {\ite_n}=0.1, {\ite_r}=0.075', ... % Lower y-coordinate position

'FontName', 'Times New Roman', 'FontSize', 16, ...

'HorizontalAlignment', 'center', ...

'VerticalAlignment', 'top', ...

'Units', 'normalized');

% Second subplot (adjust position)

subplot('Position', [0.55, 0.15, 0.35, 0.8]); % Symmetric to the first subplot

% Phase portrait of the second system (en=0.52, er=0.052)

%%%%1

k=0.8;t=0.8;cn=0.5;cr=0.2;cq=0.2;g=0.4 ;a=0.5;er=0.52;en=5.2 ;derta=0.4;theta=0.5;f=0.8;gama=0.9;%Parameter assignment

[v,y]=ode45(@(v,y) liangfang_R_xiugai(v,y,k,t,cn,cr,cq,g,a,er,en,derta,theta,f,gama),[0,50],[0.5,0.5]); %Algorithm to solve the equation, equation running time (number of steps), initial values of x and y

p=plot(v,y(:,1),'ro--','linewidth',1.5,'markersize',8);

p.MarkerIndices = 1:150:length(y(:,1));%%%%Indicates the number of markers generated; the larger the middle value, the larger the marker interval

hold on

%%%%2

k=0.8;t=0.8;cn=0.5;cr=0.2;cq=0.35;g=0.4 ;a=0.5;er=0.52;en=5.2 ;derta=0.4;theta=0.5;f=0.8;gama=0.9;%Parameter assignment

[v,y]=ode45(@(v,y) liangfang_R_xiugai(v,y,k,t,cn,cr,cq,g,a,er,en,derta,theta,f,gama),[0,50],[0.5,0.5]); %Algorithm to solve the equation, equation running time (number of steps), initial values of x and y

p=plot(v,y(:,1),'m>--','linewidth',1.5,'markersize',8);

p.MarkerIndices = 1:150:length(y(:,1));%%%%Indicates the number of markers generated; the larger the middle value, the larger the marker interval

hold on

%%%%3

k=0.8;t=0.8;cn=0.5;cr=0.2;cq=0.6;g=0.4 ;a=0.5;er=0.52;en=5.2 ;derta=0.4;theta=0.5;f=0.8;gama=0.9;%Parameter assignment

[v,y]=ode45(@(v,y) liangfang_R_xiugai(v,y,k,t,cn,cr,cq,g,a,er,en,derta,theta,f,gama),[0,50],[0.5,0.5]); %Algorithm to solve the equation, equation running time (number of steps), initial values of x and y

p=plot(v,y(:,1),'bd--','linewidth',1.5,'markersize',8);

p.MarkerIndices = 1:150:length(y(:,1));%%%%Indicates the number of markers generated; the larger the middle value, the larger the marker interval

hold on

set(gca,'XTick',0:5:50,'YTick',0:0.1:1) %%[0:axis unit length:1]

axis([0 50 0 1])%%Range of horizontal and vertical axes

xlabel('time');%%Name of horizontal axis

ylabel('x');%%Name of vertical axis

legend('{\it c_{q}}=0.2','{\it c_{q}}=0.4','{\it c_{q}}=0.6');%Legend

% Set font properties for axes and labels

set(gca, 'FontName', 'Times New Roman', 'FontSize', 16);

xlabel('\it time', 'FontName', 'Times New Roman', 'FontSize', 16); % x-axis label

ylabel('\it x', 'FontName', 'Times New Roman', 'FontSize', 16); % y-axis label

% Add title below the subplot (b)

text(0.5, -0.12, '(b) {\ite_n}=5.2, {\ite_r}=0.52', ... % Lower y-coordinate position

'FontName', 'Times New Roman', 'FontSize', 16, ...

'HorizontalAlignment', 'center', ...

'VerticalAlignment', 'top', ...

'Units', 'normalized');

axes('position',[0.6,0.25,0.1,0.2]); %Control the position of the small figure%The following is the detailed figure to be added; this position can be adjusted arbitrarily

%%%%1

k=0.8;t=0.8;cn=0.5;cr=0.2;cq=0.2;g=0.4 ;a=0.5;er=0.52;en=5.2 ;derta=0.4;theta=0.5;f=0.8;gama=0.9;%Parameter assignment

[v,y]=ode45(@(v,y) liangfang_R_xiugai(v,y,k,t,cn,cr,cq,g,a,er,en,derta,theta,f,gama),[0,50],[0.5,0.5]); %Algorithm to solve the equation, equation running time (number of steps), initial values of x and y

p=plot(v,y(:,1),'ro--','linewidth',1.5,'markersize',8);

p.MarkerIndices = 1:150:length(y(:,1));%%%%Indicates the number of markers generated; the larger the middle value, the larger the marker interval

hold on

%%%%2

k=0.8;t=0.8;cn=0.5;cr=0.2;cq=0.35;g=0.4 ;a=0.5;er=0.52;en=5.2 ;derta=0.4;theta=0.5;f=0.8;gama=0.9;%Parameter assignment

[v,y]=ode45(@(v,y) liangfang_R_xiugai(v,y,k,t,cn,cr,cq,g,a,er,en,derta,theta,f,gama),[0,50],[0.5,0.5]); %Algorithm to solve the equation, equation running time (number of steps), initial values of x and y

p=plot(v,y(:,1),'m>--','linewidth',1.5,'markersize',8);

p.MarkerIndices = 1:150:length(y(:,1));%%%%Indicates the number of markers generated; the larger the middle value, the larger the marker interval

hold on

%%%%3

k=0.8;t=0.8;cn=0.5;cr=0.2;cq=0.6;g=0.4 ;a=0.5;er=0.52;en=5.2 ;derta=0.4;theta=0.5;f=0.8;gama=0.9;%Parameter assignment

[v,y]=ode45(@(v,y) liangfang_R_xiugai(v,y,k,t,cn,cr,cq,g,a,er,en,derta,theta,f,gama),[0,50],[0.5,0.5]); %Algorithm to solve the equation, equation running time (number of steps), initial values of x and y

p=plot(v,y(:,1),'bd--','linewidth',1.5,'markersize',8);

p.MarkerIndices = 1:150:length(y(:,1));%%%%Indicates the number of markers generated; the larger the middle value, the larger the marker interval

hold on

axis([0.05 0.09 0.21 0.3 ])

Fig.5

clear; clc; % Clear workspace, clear command line

% Create figure and adjust layout

figure;

% Adjust subplot position (leave 20% space at the bottom)

subplot('Position', [0.1, 0.15, 0.35, 0.8]); % [left, bottom, width, height]

% Phase portrait of the first system (en=0.1, er=0.075)

%%%%1

k=0.8;t=0.8;cn=0.5;cr=0.2;cq=0.1;g=0.3 ;a=0.5;er=0.52;en=0.7 ;derta=0.2;theta=0.5;f=0.8;gama=0.9;%Parameter assignment

[v,y]=ode45(@(v,y) liangfang_R_xiugai(v,y,k,t,cn,cr,cq,g,a,er,en,derta,theta,f,gama),[0,50],[0.5,0.5]); %Algorithm to solve the equation, equation running time (number of steps), initial values of x and y

p=plot(v,y(:,2),'ro-','linewidth',1.5,'markersize',8);

p.MarkerIndices = 1:15:length(y(:,2));

%%Colors: y=yellow; k=black; w=white; b=blue; g=green; r=red; c=cyan; m=magenta

%%Marker styles: +, o, *, x, s=square, d=diamond, v=down triangle, >=right triangle, <=left triangle, p=pentagram, h=hexagram, none=no marker

%Line styles: -solid, --dashed, -.-dash-dot, .dotted

%linewidth = line width, markersize = marker size, MarkerIndices = marker interval, larger middle value in 1:1:length(t) means larger marker interval

hold on

%%%%2

k=0.8;t=0.8;cn=0.5;cr=0.2;cq=0.4;g=0.3 ;a=0.5;er=0.52;en=0.7 ;derta=0.2;theta=0.5;f=0.8;gama=0.9;%Parameter assignment

[v,y]=ode45(@(v,y) liangfang_R_xiugai(v,y,k,t,cn,cr,cq,g,a,er,en,derta,theta,f,gama),[0,50],[0.5,0.5]); %Algorithm to solve the equation, equation running time (number of steps), initial values of x and y

p=plot(v,y(:,2),'m>-','linewidth',1.5,'markersize',8);

p.MarkerIndices = 1:15:length(y(:,2));%%%%Indicates the number of markers generated; the larger the middle value, the larger the marker interval

hold on

%%%%3

k=0.8;t=0.8;cn=0.5;cr=0.2;cq=0.8;g=0.3 ;a=0.5;er=0.52;en=0.7 ;derta=0.2;theta=0.5;f=0.8;gama=0.9;%Parameter assignment

[v,y]=ode45(@(v,y) liangfang_R_xiugai(v,y,k,t,cn,cr,cq,g,a,er,en,derta,theta,f,gama),[0,50],[0.5,0.5]); %Algorithm to solve the equation, equation running time (number of steps), initial values of x and y

p=plot(v,y(:,2),'bd-','linewidth',1.5,'markersize',8);

p.MarkerIndices = 1:15:length(y(:,2));%%%%Indicates the number of markers generated; the larger the middle value, the larger the marker interval

hold on

set(gca,'XTick',0:5:50,'YTick',0:0.1:1) %%[0:axis unit length:1]

axis([0 50 0 1])%%Range of horizontal and vertical axes

xlabel('time');%%Name of horizontal axis

ylabel('x');%%Name of vertical axis

set(gca,'XTick',0:5:50,'YTick',0:0.1:1) %%[0:axis unit length:1]

axis([0 50 0 1])%%Range of horizontal and vertical axes

xlabel('time');%%Name of horizontal axis

ylabel('y');%%Name of vertical axis

legend('{\it c_{q}}=0.2','{\it c_{q}}=0.4','{\it c_{q}}=0.6');%Legend

% Set font properties for axes and labels

set(gca, 'FontName', 'Times New Roman', 'FontSize', 16);

xlabel('\it time', 'FontName', 'Times New Roman', 'FontSize', 16); % x-axis label

ylabel('\it y', 'FontName', 'Times New Roman', 'FontSize', 16); % y-axis label

% Add title below the subplot (a)

text(0.5, -0.12, '(a) {\ite_n}=0.1, {\ite_r}=0.075', ... % Lower y-coordinate position

'FontName', 'Times New Roman', 'FontSize', 16, ...

'HorizontalAlignment', 'center', ...

'VerticalAlignment', 'top', ...

'Units', 'normalized');

% Second subplot (adjust position)

subplot('Position', [0.55, 0.15, 0.35, 0.8]); % Symmetric to the first subplot

% Phase portrait of the second system (en=0.52, er=0.052)

%%%%1

k=0.8;t=0.8;cn=0.5;cr=0.2;cq=0.2;g=0.3 ;a=0.5;er=0.52;en=5.2 ;derta=0.2;theta=0.5;f=0.8;gama=0.9;%Parameter assignment

[v,y]=ode45(@(v,y) liangfang_R_xiugai(v,y,k,t,cn,cr,cq,g,a,er,en,derta,theta,f,gama),[0,50],[0.5,0.5]); %Algorithm to solve the equation, equation running time (number of steps), initial values of x and y

p=plot(v,y(:,2),'ro-','linewidth',1.5,'markersize',8);

p.MarkerIndices = 1:50:length(y(:,2));

%%Colors: y=yellow; k=black; w=white; b=blue; g=green; r=red; c=cyan; m=magenta

%%Marker styles: +, o, *, x, s=square, d=diamond, v=down triangle, >=right triangle, <=left triangle, p=pentagram, h=hexagram, none=no marker

%Line styles: -solid, --dashed, -.-dash-dot, .dotted

%linewidth = line width, markersize = marker size, MarkerIndices = marker interval, larger middle value in 1:1:length(t) means larger marker interval

hold on

%%%%2

k=0.8;t=0.8;cn=0.5;cr=0.2;cq=0.35;g=0.3 ;a=0.5;er=0.52;en=5.2 ;derta=0.2;theta=0.5;f=0.8;gama=0.9;%Parameter assignment

[v,y]=ode45(@(v,y) liangfang_R_xiugai(v,y,k,t,cn,cr,cq,g,a,er,en,derta,theta,f,gama),[0,50],[0.5,0.5]); %Algorithm to solve the equation, equation running time (number of steps), initial values of x and y

p=plot(v,y(:,2),'m>-','linewidth',1.5,'markersize',8);

p.MarkerIndices = 1:50:length(y(:,2));%%%%Indicates the number of markers generated; the larger the middle value, the larger the marker interval

hold on

%%%%3

k=0.8;t=0.8;cn=0.5;cr=0.2;cq=0.6;g=0.3 ;a=0.5;er=0.52;en=5.2 ;derta=0.2;theta=0.5;f=0.8;gama=0.9;%Parameter assignment

[v,y]=ode45(@(v,y) liangfang_R_xiugai(v,y,k,t,cn,cr,cq,g,a,er,en,derta,theta,f,gama),[0,50],[0.5,0.5]); %Algorithm to solve the equation, equation running time (number of steps), initial values of x and y

p=plot(v,y(:,2),'bd-','linewidth',1.5,'markersize',8);

p.MarkerIndices = 1:50:length(y(:,2));%%%%Indicates the number of markers generated; the larger the middle value, the larger the marker interval

hold on

set(gca,'XTick',0:5:50,'YTick',0:0.1:1) %%[0:axis unit length:1]

axis([0 50 0 1])%%Range of horizontal and vertical axes

xlabel('time');%%Name of horizontal axis

ylabel('x');%%Name of vertical axis

set(gca,'XTick',0:5:50,'YTick',0:0.1:1) %%[0:axis unit length:1]

axis([0 50 0 1])%%Range of horizontal and vertical axes

xlabel('time');%%Name of horizontal axis

ylabel('y');%%Name of vertical axis

legend('{\it c_{q}}=0.2','{\it c_{q}}=0.4','{\it c_{q}}=0.6');%Legend

% Set font properties for axes and labels

set(gca, 'FontName', 'Times New Roman', 'FontSize', 16);

xlabel('\it time', 'FontName', 'Times New Roman', 'FontSize', 16); % x-axis label

ylabel('\it y', 'FontName', 'Times New Roman', 'FontSize', 16); % y-axis label

% Add title below the subplot (b)

text(0.5, -0.12, '(b) {\ite_n}=5.2, {\ite_r}=0.52', ... % Lower y-coordinate position

'FontName', 'Times New Roman', 'FontSize', 16, ...

'HorizontalAlignment', 'center', ...

'VerticalAlignment', 'top', ...

'Units', 'normalized');

Fig.6

clear; clc; % Clear workspace, clear command line

% Create figure and adjust layout

figure;

% Adjust subplot position (leave 20% space at the bottom)

subplot('Position', [0.1, 0.15, 0.35, 0.8]); % [left, bottom, width, height]

% Phase portrait of the first system (en=0.1, er=0.075)

%%%%1

k=0.8;t=0;cn=0.5;cr=0.2;cq=0.5;g=0.3;a=0.5;er=0.52;en=0.7 ;derta=0.4;theta=0.5;f=0.8;gama=0.9;%Parameter assignment

[v,y]=ode45(@(v,y) liangfang_R_xiugai(v,y,k,t,cn,cr,cq,g,a,er,en,derta,theta,f,gama),[0,50],[0.5,0.5]); %Algorithm to solve the equation, equation running time (number of steps), initial values of x and y

p=plot(v,y(:,1),'ro--','linewidth',1.5,'markersize',8);

p.MarkerIndices = 1:15:length(y(:,1));%%%%Indicates the number of markers generated; the larger the middle value, the larger the marker interval

hold on

%%%%2

k=0.8;t=0.5;cn=0.5;cr=0.2;cq=0.2;g=0.4 ;a=0.5;er=0.52;en=0.7 ;derta=0.4;theta=0.5;f=0.8;gama=0.9;%Parameter assignment

[v,y]=ode45(@(v,y) liangfang_R_xiugai(v,y,k,t,cn,cr,cq,g,a,er,en,derta,theta,f,gama),[0,50],[0.5,0.5]); %Algorithm to solve the equation, equation running time (number of steps), initial values of x and y

p=plot(v,y(:,1),'m>--','linewidth',1.5,'markersize',8);

p.MarkerIndices = 1:15:length(y(:,1));%%%%Indicates the number of markers generated; the larger the middle value, the larger the marker interval

hold on

%%%%3

k=0.8;t=0.9;cn=0.5;cr=0.2;cq=0.2;g=0.4 ;a=0.5;er=0.52;en=0.7 ;derta=0.4;theta=0.5;f=0.8;gama=0.9;%Parameter assignment

[v,y]=ode45(@(v,y) liangfang_R_xiugai(v,y,k,t,cn,cr,cq,g,a,er,en,derta,theta,f,gama),[0,50],[0.5,0.5]); %Algorithm to solve the equation, equation running time (number of steps), initial values of x and y

p=plot(v,y(:,1),'bd--','linewidth',1.5,'markersize',8);

p.MarkerIndices = 1:15:length(y(:,1));%%%%Indicates the number of markers generated; the larger the middle value, the larger the marker interval

hold on

set(gca,'XTick',0:5:50,'YTick',0:0.1:1) %%[0:axis unit length:1]

axis([0 50 0 1])%%Range of horizontal and vertical axes

xlabel('time');%%Name of horizontal axis

ylabel('x');%%Name of vertical axis

legend('\it t=0', '\it t=0.5', '\it t=0.9','FontSize', 18); %%%Legend

% Set font properties for axes and labels

set(gca, 'FontName', 'Times New Roman', 'FontSize', 16);

xlabel('\it time', 'FontName', 'Times New Roman', 'FontSize', 16); % x-axis label

ylabel('\it x', 'FontName', 'Times New Roman', 'FontSize', 16); % y-axis label

% Add title below the subplot (a)

text(0.5, -0.12, '(a) {\ite_n}=0.1, {\ite_r}=0.075', ... % Lower y-coordinate position

'FontName', 'Times New Roman', 'FontSize', 16, ...

'HorizontalAlignment', 'center', ...

'VerticalAlignment', 'top', ...

'Units', 'normalized');

% Second subplot (adjust position)

subplot('Position', [0.55, 0.15, 0.35, 0.8]); % Symmetric to the first subplot

% Phase portrait of the second system (en=0.52, er=0.052)

%%%%1

k=0.8;t=0;cn=0.5;cr=0.2;cq=0.2;g=0.3;a=0.5;er=0.52;en=5.2 ;derta=0.4;theta=0.5;f=0.8;gama=0.9;%Parameter assignment

[v,y]=ode45(@(v,y) liangfang_R_xiugai(v,y,k,t,cn,cr,cq,g,a,er,en,derta,theta,f,gama),[0,50],[0.5,0.5]); %Algorithm to solve the equation, equation running time (number of steps), initial values of x and y

p=plot(v,y(:,1),'ro--','linewidth',1.5,'markersize',8);

p.MarkerIndices = 1:150:length(y(:,1));%%%%Indicates the number of markers generated; the larger the middle value, the larger the marker interval

hold on

%%%%2

k=0.8;t=0.5;cn=0.5;cr=0.2;cq=0.2;g=0.4 ;a=0.5;er=0.52;en=5.2 ;derta=0.4;theta=0.5;f=0.8;gama=0.9;%Parameter assignment

[v,y]=ode45(@(v,y) liangfang_R_xiugai(v,y,k,t,cn,cr,cq,g,a,er,en,derta,theta,f,gama),[0,50],[0.5,0.5]); %Algorithm to solve the equation, equation running time (number of steps), initial values of x and y

p=plot(v,y(:,1),'m>--','linewidth',1.5,'markersize',8);

p.MarkerIndices = 1:150:length(y(:,1));%%%%Indicates the number of markers generated; the larger the middle value, the larger the marker interval

hold on

%%%%3

k=0.8;t=0.9;cn=0.5;cr=0.2;cq=0.2;g=0.4 ;a=0.5;er=0.52;en=5.2 ;derta=0.4;theta=0.5;f=0.8;gama=0.9;%Parameter assignment

[v,y]=ode45(@(v,y) liangfang_R_xiugai(v,y,k,t,cn,cr,cq,g,a,er,en,derta,theta,f,gama),[0,50],[0.5,0.5]); %Algorithm to solve the equation, equation running time (number of steps), initial values of x and y

p=plot(v,y(:,1),'bd--','linewidth',1.5,'markersize',8);

p.MarkerIndices = 1:150:length(y(:,1));%%%%Indicates the number of markers generated; the larger the middle value, the larger the marker interval

hold on

set(gca,'XTick',0:5:50,'YTick',0:0.1:1) %%[0:axis unit length:1]

axis([0 50 0 1])%%Range of horizontal and vertical axes

xlabel('time');%%Name of horizontal axis

ylabel('x');%%Name of vertical axis

legend('\it t=0', '\it t=0.5', '\it t=0.9','FontSize', 18); %%%Legend

% Set font properties for axes and labels

set(gca, 'FontName', 'Times New Roman', 'FontSize', 16);

xlabel('\it time', 'FontName', 'Times New Roman', 'FontSize', 16); % x-axis label

ylabel('\it x', 'FontName', 'Times New Roman', 'FontSize', 16); % y-axis label

% Add title below the subplot (b)

text(0.5, -0.12, '(b) {\ite_n}=5.2, {\ite_r}=0.52', ... % Lower y-coordinate position

'FontName', 'Times New Roman', 'FontSize', 16, ...

'HorizontalAlignment', 'center', ...

'VerticalAlignment', 'top', ...

'Units', 'normalized');

axes('position',[0.6,0.25,0.1,0.2]); %Control the position of the small figure%The following is the detailed figure to be added; this position can be adjusted arbitrarily

%%%%1

k=0.8;t=0;cn=0.5;cr=0.2;cq=0.2;g=0.3;a=0.5;er=0.52;en=5.2 ;derta=0.4;theta=0.5;f=0.8;gama=0.9;%Parameter assignment

[v,y]=ode45(@(v,y) liangfang_R_xiugai(v,y,k,t,cn,cr,cq,g,a,er,en,derta,theta,f,gama),[0,50],[0.5,0.5]); %Algorithm to solve the equation, equation running time (number of steps), initial values of x and y

p=plot(v,y(:,1),'ro--','linewidth',1.5,'markersize',8);

p.MarkerIndices = 1:150:length(y(:,1));%%%%Indicates the number of markers generated; the larger the middle value, the larger the marker interval

hold on

%%%%2

k=0.8;t=0.5;cn=0.5;cr=0.2;cq=0.2;g=0.4 ;a=0.5;er=0.52;en=5.2 ;derta=0.4;theta=0.5;f=0.8;gama=0.9;%Parameter assignment

[v,y]=ode45(@(v,y) liangfang_R_xiugai(v,y,k,t,cn,cr,cq,g,a,er,en,derta,theta,f,gama),[0,50],[0.5,0.5]); %Algorithm to solve the equation, equation running time (number of steps), initial values of x and y

p=plot(v,y(:,1),'m>--','linewidth',1.5,'markersize',8);

p.MarkerIndices = 1:150:length(y(:,1));%%%%Indicates the number of markers generated; the larger the middle value, the larger the marker interval

hold on

%%%%3

k=0.8;t=0.9;cn=0.5;cr=0.2;cq=0.2;g=0.4 ;a=0.5;er=0.52;en=5.2 ;derta=0.4;theta=0.5;f=0.8;gama=0.9;%Parameter assignment

[v,y]=ode45(@(v,y) liangfang_R_xiugai(v,y,k,t,cn,cr,cq,g,a,er,en,derta,theta,f,gama),[0,50],[0.5,0.5]); %Algorithm to solve the equation, equation running time (number of steps), initial values of x and y

p=plot(v,y(:,1),'bd--','linewidth',1.5,'markersize',8);

p.MarkerIndices = 1:150:length(y(:,1));%%%%Indicates the number of markers generated; the larger the middle value, the larger the marker interval

hold on

axis([0 0.04 0.45 0.55])

Fig.7

clear; clc; % Clear workspace, clear command line

% Create figure and adjust layout

figure;

% Adjust subplot position (leave 20% space at the bottom)

subplot('Position', [0.1, 0.15, 0.35, 0.8]); % [left, bottom, width, height]

% Phase portrait of the first system (en=0.1, er=0.075)

%%%%1

k=0.8;t=0;cn=0.5;cr=0.2;cq=0.2;g=0.3 ;a=0.5;er=0.52;en=0.7 ;derta=0.2;theta=0.5;f=0.8;gama=0.9;%Parameter assignment

[v,y]=ode45(@(v,y) liangfang_R_xiugai(v,y,k,t,cn,cr,cq,g,a,er,en,derta,theta,f,gama),[0,50],[0.5,0.5]); %Algorithm to solve the equation, equation running time (number of steps), initial values of x and y

p=plot(v,y(:,2),'ro-','linewidth',1.5,'markersize',8);

p.MarkerIndices = 1:10:length(y(:,2));

%%Colors: y=yellow; k=black; w=white; b=blue; g=green; r=red; c=cyan; m=magenta

%%Marker styles: +, o, *, x, s=square, d=diamond, v=down triangle, >=right triangle, <=left triangle, p=pentagram, h=hexagram, none=no marker

%Line styles: -solid, --dashed, -.-dash-dot, .dotted

%linewidth = line width, markersize = marker size, MarkerIndices = marker interval, larger middle value in 1:1:length(t) means larger marker interval

hold on

%%%%2

k=0.8;t=0.5;cn=0.5;cr=0.2;cq=0.2;g=0.3 ;a=0.5;er=0.52;en=0.7 ;derta=0.2;theta=0.5;f=0.8;gama=0.9;%Parameter assignment

[v,y]=ode45(@(v,y) liangfang_R_xiugai(v,y,k,t,cn,cr,cq,g,a,er,en,derta,theta,f,gama),[0,50],[0.5,0.5]); %Algorithm to solve the equation, equation running time (number of steps), initial values of x and y

p=plot(v,y(:,2),'m>-','linewidth',1.5,'markersize',8);

p.MarkerIndices = 1:10:length(y(:,2));%%%%Indicates the number of markers generated; the larger the middle value, the larger the marker interval

hold on

%%%%3

k=0.8;t=0.9;cn=0.5;cr=0.2;cq=0.2;g=0.3 ;a=0.5;er=0.52;en=0.7 ;derta=0.2;theta=0.5;f=0.8;gama=0.9;%Parameter assignment

[v,y]=ode45(@(v,y) liangfang_R_xiugai(v,y,k,t,cn,cr,cq,g,a,er,en,derta,theta,f,gama),[0,50],[0.5,0.5]); %Algorithm to solve the equation, equation running time (number of steps), initial values of x and y

p=plot(v,y(:,2),'bd-','linewidth',1.5,'markersize',8);

p.MarkerIndices = 1:10:length(y(:,2));%%%%Indicates the number of markers generated; the larger the middle value, the larger the marker interval

hold on

set(gca,'XTick',0:5:50,'YTick',0:0.1:1) %%[0:axis unit length:1]

axis([0 50 0 1])%%Range of horizontal and vertical axes

xlabel('time');%%Name of horizontal axis

ylabel('x');%%Name of vertical axis

set(gca,'XTick',0:5:50,'YTick',0:0.1:1) %%[0:axis unit length:1]

axis([0 50 0 1])%%Range of horizontal and vertical axes

xlabel('time');%%Name of horizontal axis

ylabel('y');%%Name of vertical axis

legend('\it t=0', '\it t=0.5', '\it t=0.9','FontSize', 18); %%%Legend

% Set font properties for axes and labels

set(gca, 'FontName', 'Times New Roman', 'FontSize', 16);

xlabel('\it time', 'FontName', 'Times New Roman', 'FontSize', 16); % x-axis label

ylabel('\it y', 'FontName', 'Times New Roman', 'FontSize', 16); % y-axis label

% Add title below the subplot (a)

text(0.5, -0.12, '(a) {\ite_n}=0.1, {\ite_r}=0.075', ... % Lower y-coordinate position

'FontName', 'Times New Roman', 'FontSize', 16, ...

'HorizontalAlignment', 'center', ...

'VerticalAlignment', 'top', ...

'Units', 'normalized');

% Second subplot (adjust position)

subplot('Position', [0.55, 0.15, 0.35, 0.8]); % Symmetric to the first subplot

% Phase portrait of the second system (en=0.52, er=0.052)

%%%%1

k=0.8;t=0;cn=0.5;cr=0.2;cq=0.2;g=0.5 ;a=0.5;er=0.52;en=5.2 ;derta=0.2;theta=0.5;f=0.8;gama=0.9;%Parameter assignment

[v,y]=ode45(@(v,y) liangfang_R_xiugai(v,y,k,t,cn,cr,cq,g,a,er,en,derta,theta,f,gama),[0,50],[0.5,0.5]); %Algorithm to solve the equation, equation running time (number of steps), initial values of x and y

p=plot(v,y(:,2),'ro-','linewidth',1.5,'markersize',8);

p.MarkerIndices = 1:15:length(y(:,2));

%%Colors: y=yellow; k=black; w=white; b=blue; g=green; r=red; c=cyan; m=magenta

%%Marker styles: +, o, *, x, s=square, d=diamond, v=down triangle, >=right triangle, <=left triangle, p=pentagram, h=hexagram, none=no marker

%Line styles: -solid, --dashed, -.-dash-dot, .dotted

%linewidth = line width, markersize = marker size, MarkerIndices = marker interval, larger middle value in 1:1:length(t) means larger marker interval

hold on

%%%%2

k=0.8;t=0.5;cn=0.5;cr=0.2;cq=0.2;g=0.5 ;a=0.5;er=0.52;en=5.2 ;derta=0.2;theta=0.5;f=0.8;gama=0.9;%Parameter assignment

[v,y]=ode45(@(v,y) liangfang_R_xiugai(v,y,k,t,cn,cr,cq,g,a,er,en,derta,theta,f,gama),[0,50],[0.5,0.5]); %Algorithm to solve the equation, equation running time (number of steps), initial values of x and y

p=plot(v,y(:,2),'m>-','linewidth',1.5,'markersize',8);

p.MarkerIndices = 1:50:length(y(:,2));%%%%Indicates the number of markers generated; the larger the middle value, the larger the marker interval

hold on

%%%%3

k=0.8;t=0.9;cn=0.5;cr=0.2;cq=0.2;g=0.5 ;a=0.5;er=0.52;en=5.2 ;derta=0.2;theta=0.5;f=0.8;gama=0.9;%Parameter assignment

[v,y]=ode45(@(v,y) liangfang_R_xiugai(v,y,k,t,cn,cr,cq,g,a,er,en,derta,theta,f,gama),[0,50],[0.5,0.5]); %Algorithm to solve the equation, equation running time (number of steps), initial values of x and y

p=plot(v,y(:,2),'bd-','linewidth',1.5,'markersize',8);

p.MarkerIndices = 1:100:length(y(:,2));%%%%Indicates the number of markers generated; the larger the middle value, the larger the marker interval

hold on

set(gca,'XTick',0:5:50,'YTick',0:0.1:1) %%[0:axis unit length:1]

axis([0 50 0 1])%%Range of horizontal and vertical axes

xlabel('time');%%Name of horizontal axis

ylabel('x');%%Name of vertical axis

set(gca,'XTick',0:5:50,'YTick',0:0.1:1) %%[0:axis unit length:1]

axis([0 50 0 1])%%Range of horizontal and vertical axes

xlabel('time');%%Name of horizontal axis

ylabel('y');%%Name of vertical axis

legend('\it t=0', '\it t=0.5', '\it t=0.9','FontSize', 18); %%%Legend

% Set font properties for axes and labels

set(gca, 'FontName', 'Times New Roman', 'FontSize', 16);

xlabel('\it time', 'FontName', 'Times New Roman', 'FontSize', 16); % x-axis label

ylabel('\it y', 'FontName', 'Times New Roman', 'FontSize', 16); % y-axis label

% Add title below the subplot (b)

text(0.5, -0.12, '(b) {\ite_n}=5.2, {\ite_r}=0.52', ... % Lower y-coordinate position

'FontName', 'Times New Roman', 'FontSize', 16, ...

'HorizontalAlignment', 'center', ...

'VerticalAlignment', 'top', ...

'Units', 'normalized');

Fig.8

clear; clc; % Clear workspace, clear command line

% Create figure and adjust layout

figure;

% Adjust subplot position (leave 20% space at the bottom)

subplot('Position', [0.1, 0.15, 0.35, 0.8]); % [left, bottom, width, height]

% Phase portrait of the first system (en=0.1, er=0.075)

%%%%1

k=0.8;t=0.8;cn=0.5;cr=0.2;cq=0.2;g=0.4 ;a=0.5;er=0.52;en=0.7 ;derta=0.4;theta=0.5;f=0.8;gama=0;%Parameter assignment

[v,y]=ode45(@(v,y) liangfang_R_xiugai(v,y,k,t,cn,cr,cq,g,a,er,en,derta,theta,f,gama),[0,50],[0.5,0.5]); %Algorithm to solve the equation, equation running time (number of steps), initial values of x and y

p=plot(v,y(:,1),'ro--','linewidth',1.5,'markersize',8);

p.MarkerIndices = 1:15:length(y(:,1));%%%%Indicates the number of markers generated; the larger the middle value, the larger the marker interval

hold on

%%%%2

k=0.8;t=0.8;cn=0.5;cr=0.2;cq=0.2;g=0.4 ;a=0.5;er=0.52;en=0.7 ;derta=0.4;theta=0.5;f=0.8;gama=0.5;%Parameter assignment

[v,y]=ode45(@(v,y) liangfang_R_xiugai(v,y,k,t,cn,cr,cq,g,a,er,en,derta,theta,f,gama),[0,50],[0.5,0.5]); %Algorithm to solve the equation, equation running time (number of steps), initial values of x and y

p=plot(v,y(:,1),'m>--','linewidth',1.5,'markersize',8);

p.MarkerIndices = 1:15:length(y(:,1));%%%%Indicates the number of markers generated; the larger the middle value, the larger the marker interval

hold on

%%%%3

k=0.8;t=0.8;cn=0.5;cr=0.2;cq=0.2;g=0.4 ;a=0.5;er=0.52;en=0.7 ;derta=0.4;theta=0.5;f=0.8;gama=0.9;%Parameter assignment

[v,y]=ode45(@(v,y) liangfang_R_xiugai(v,y,k,t,cn,cr,cq,g,a,er,en,derta,theta,f,gama),[0,50],[0.5,0.5]); %Algorithm to solve the equation, equation running time (number of steps), initial values of x and y

p=plot(v,y(:,1),'bd--','linewidth',1.5,'markersize',8);

p.MarkerIndices = 1:15:length(y(:,1));%%%%Indicates the number of markers generated; the larger the middle value, the larger the marker interval

hold on

set(gca,'XTick',0:5:50,'YTick',0:0.1:1) %%[0:axis unit length:1]

axis([0 50 0 1])%%Range of horizontal and vertical axes

xlabel('time');%%Name of horizontal axis

ylabel('x');%%Name of vertical axis

legend('\gamma=0', '\gamma=0.5', '\gamma=0.9','FontSize', 18); %%%Legend

% Set font properties for axes and labels

set(gca, 'FontName', 'Times New Roman', 'FontSize', 16);

xlabel('\it time', 'FontName', 'Times New Roman', 'FontSize', 16); % x-axis label

ylabel('\it x', 'FontName', 'Times New Roman', 'FontSize', 16); % y-axis label

% Add title below the subplot (a)

text(0.5, -0.12, '(a) {\ite_n}=0.1, {\ite_r}=0.075', ... % Lower y-coordinate position

'FontName', 'Times New Roman', 'FontSize', 16, ...

'HorizontalAlignment', 'center', ...

'VerticalAlignment', 'top', ...

'Units', 'normalized');

% Second subplot (adjust position)

subplot('Position', [0.55, 0.15, 0.35, 0.8]); % Symmetric to the first subplot

% Phase portrait of the second system (en=0.52, er=0.052)

%%%%1

k=0.8;t=0.8;cn=0.5;cr=0.2;cq=0.2;g=0.3 ;a=0.5;er=0.52;en=1.3 ;derta=0.3;theta=0.9;f=0.8;gama=0;%Parameter assignment

[v,y]=ode45(@(v,y) liangfang_R_xiugai(v,y,k,t,cn,cr,cq,g,a,er,en,derta,theta,f,gama),[0,50],[0.5,0.5]); %Algorithm to solve the equation, equation running time (number of steps), initial values of x and y

p=plot(v,y(:,1),'ro--','linewidth',1.5,'markersize',8);

p.MarkerIndices = 1:10:length(y(:,1));%%%%Indicates the number of markers generated; the larger the middle value, the larger the marker interval

hold on

%%%%2

k=0.8;t=0.8;cn=0.5;cr=0.2;cq=0.2;g=0.3 ;a=0.5;er=0.52;en=1.3 ;derta=0.3;theta=0.1;f=0.8;gama=0.5;%Parameter assignment

[v,y]=ode45(@(v,y) liangfang_R_xiugai(v,y,k,t,cn,cr,cq,g,a,er,en,derta,theta,f,gama),[0,50],[0.5,0.5]); %Algorithm to solve the equation, equation running time (number of steps), initial values of x and y

p=plot(v,y(:,1),'m>--','linewidth',1.5,'markersize',8);

p.MarkerIndices = 1:10:length(y(:,1));%%%%Indicates the number of markers generated; the larger the middle value, the larger the marker interval

hold on

%%%%3

k=0.8;t=0.8;cn=0.5;cr=0.2;cq=0.2;g=0.3 ;a=0.5;er=0.52;en=1.3 ;derta=0.3;theta=0.1;f=0.8;gama=0.9;%Parameter assignment

[v,y]=ode45(@(v,y) liangfang_R_xiugai(v,y,k,t,cn,cr,cq,g,a,er,en,derta,theta,f,gama),[0,50],[0.5,0.5]); %Algorithm to solve the equation, equation running time (number of steps), initial values of x and y

p=plot(v,y(:,1),'bd--','linewidth',1.5,'markersize',8);

p.MarkerIndices = 1:10:length(y(:,1));%%%%Indicates the number of markers generated; the larger the middle value, the larger the marker interval

hold on

set(gca,'XTick',0:5:50,'YTick',0:0.1:1) %%[0:axis unit length:1]

axis([0 50 0 1])%%Range of horizontal and vertical axes

xlabel('time');%%Name of horizontal axis

ylabel('x');%%Name of vertical axis

legend('\gamma=0', '\gamma=0.5', '\gamma=0.9','FontSize', 18); %%%Legend

% Set font properties for axes and labels

set(gca, 'FontName', 'Times New Roman', 'FontSize', 16);

xlabel('\it time', 'FontName', 'Times New Roman', 'FontSize', 16); % x-axis label

ylabel('\it x', 'FontName', 'Times New Roman', 'FontSize', 16); % y-axis label

% Add title below the subplot (b)

text(0.5, -0.12, '(b) {\ite_n}=5.2, {\ite_r}=0.52', ... % Lower y-coordinate position

'FontName', 'Times New Roman', 'FontSize', 16, ...

'HorizontalAlignment', 'center', ...

'VerticalAlignment', 'top', ...

'Units', 'normalized');

Fig.9

clear; clc; % Clear workspace, clear command line

% Create figure and adjust layout

figure;

% Adjust subplot position (leave 20% space at the bottom)

subplot('Position', [0.1, 0.15, 0.35, 0.8]); % [left, bottom, width, height]

% Phase portrait of the first system (en=0.1, er=0.075)

%%%%1

k=0.8;t=0.8;cn=0.5;cr=0.2;cq=0.2;g=0.3 ;a=0.5;er=0.52;en=0.7 ;derta=0.2;theta=0.5;f=0.8;gama=0;%Parameter assignment

[v,y]=ode45(@(v,y) liangfang_R_xiugai(v,y,k,t,cn,cr,cq,g,a,er,en,derta,theta,f,gama),[0,50],[0.5,0.5]); %Algorithm to solve the equation, equation running time (number of steps), initial values of x and y

p=plot(v,y(:,2),'ro-','linewidth',1.5,'markersize',8);

p.MarkerIndices = 1:5:length(y(:,2));

%%Colors: y=yellow; k=black; w=white; b=blue; g=green; r=red; c=cyan; m=magenta

%%Marker styles: +, o, *, x, s=square, d=diamond, v=down triangle, >=right triangle, <=left triangle, p=pentagram, h=hexagram, none=no marker

%Line styles: -solid, --dashed, -.-dash-dot, .dotted

%linewidth = line width, markersize = marker size, MarkerIndices = marker interval, larger middle value in 1:1:length(t) means larger marker interval

hold on

%%%%2

k=0.8;t=0.8;cn=0.5;cr=0.2;cq=0.2;g=0.3 ;a=0.5;er=0.52;en=0.7 ;derta=0.2;theta=0.5;f=0.8;gama=0.5;%Parameter assignment

[v,y]=ode45(@(v,y) liangfang_R_xiugai(v,y,k,t,cn,cr,cq,g,a,er,en,derta,theta,f,gama),[0,50],[0.5,0.5]); %Algorithm to solve the equation, equation running time (number of steps), initial values of x and y

p=plot(v,y(:,2),'m>-','linewidth',1.5,'markersize',8);

p.MarkerIndices = 1:5:length(y(:,2));%%%%Indicates the number of markers generated; the larger the middle value, the larger the marker interval

hold on

%%%%3

k=0.8;t=0.8;cn=0.5;cr=0.2;cq=0.2;g=0.3 ;a=0.5;er=0.52;en=0.7 ;derta=0.2;theta=0.5;f=0.8;gama=0.9;%Parameter assignment

[v,y]=ode45(@(v,y) liangfang_R_xiugai(v,y,k,t,cn,cr,cq,g,a,er,en,derta,theta,f,gama),[0,50],[0.5,0.5]); %Algorithm to solve the equation, equation running time (number of steps), initial values of x and y

p=plot(v,y(:,2),'bd-','linewidth',1.5,'markersize',8);

p.MarkerIndices = 1:5:length(y(:,2));%%%%Indicates the number of markers generated; the larger the middle value, the larger the marker interval

hold on

set(gca,'XTick',0:5:50,'YTick',0:0.1:1) %%[0:axis unit length:1]

axis([0 50 0 1])%%Range of horizontal and vertical axes

xlabel('time');%%Name of horizontal axis

ylabel('x');%%Name of vertical axis

set(gca,'XTick',0:5:50,'YTick',0:0.1:1) %%[0:axis unit length:1]

axis([0 50 0 1])%%Range of horizontal and vertical axes

xlabel('time');%%Name of horizontal axis

ylabel('y');%%Name of vertical axis

legend('\gamma=0', '\gamma=0.5', '\gamma=0.9','FontSize', 18); %%%Legend

% Set font properties for axes and labels

set(gca, 'FontName', 'Times New Roman', 'FontSize', 16);

xlabel('\it time', 'FontName', 'Times New Roman', 'FontSize', 16); % x-axis label

ylabel('\it y', 'FontName', 'Times New Roman', 'FontSize', 16); % y-axis label

% Add title below the subplot (a)

text(0.5, -0.12, '(a) {\ite_n}=0.1, {\ite_r}=0.075', ... % Lower y-coordinate position

'FontName', 'Times New Roman', 'FontSize', 16, ...

'HorizontalAlignment', 'center', ...

'VerticalAlignment', 'top', ...

'Units', 'normalized');

% Second subplot (adjust position)

subplot('Position', [0.55, 0.15, 0.35, 0.8]); % Symmetric to the first subplot

% Phase portrait of the second system (en=0.52, er=0.052)

%%%%1

k=0.8;t=0.8;cn=0.5;cr=0.2;cq=0.2;g=0.35 ;a=0.5;er=0.52;en=5.2 ;derta=0.2;theta=0.3;f=0.8;gama=0;%Parameter assignment

[v,y]=ode45(@(v,y) liangfang_R_xiugai(v,y,k,t,cn,cr,cq,g,a,er,en,derta,theta,f,gama),[0,50],[0.5,0.5]); %Algorithm to solve the equation, equation running time (number of steps), initial values of x and y

p=plot(v,y(:,2),'ro-','linewidth',1.5,'markersize',8);

p.MarkerIndices = 1:50:length(y(:,2));

%%Colors: y=yellow; k=black; w=white; b=blue; g=green; r=red; c=cyan; m=magenta

%%Marker styles: +, o, *, x, s=square, d=diamond, v=down triangle, >=right triangle, <=left triangle, p=pentagram, h=hexagram, none=no marker

%Line styles: -solid, --dashed, -.-dash-dot, .dotted

%linewidth = line width, markersize = marker size, MarkerIndices = marker interval, larger middle value in 1:1:length(t) means larger marker interval

hold on

%%%%2

k=0.8;t=0.8;cn=0.5;cr=0.2;cq=0.2;g=0.35 ;a=0.5;er=0.52;en=5.2 ;derta=0.2;theta=0.3;f=0.8;gama=0.5;%Parameter assignment

[v,y]=ode45(@(v,y) liangfang_R_xiugai(v,y,k,t,cn,cr,cq,g,a,er,en,derta,theta,f,gama),[0,50],[0.5,0.5]); %Algorithm to solve the equation, equation running time (number of steps), initial values of x and y

p=plot(v,y(:,2),'m>-','linewidth',1.5,'markersize',8);

p.MarkerIndices = 1:50:length(y(:,2));%%%%Indicates the number of markers generated; the larger the middle value, the larger the marker interval

hold on

%%%%3

k=0.8;t=0.8;cn=0.5;cr=0.2;cq=0.2;g=0.35;a=0.5;er=0.52;en=5.2 ;derta=0.2;theta=0.3;f=0.8;gama=0.9;%Parameter assignment

[v,y]=ode45(@(v,y) liangfang_R_xiugai(v,y,k,t,cn,cr,cq,g,a,er,en,derta,theta,f,gama),[0,50],[0.5,0.5]); %Algorithm to solve the equation, equation running time (number of steps), initial values of x and y

p=plot(v,y(:,2),'bd-','linewidth',1.5,'markersize',8);

p.MarkerIndices = 1:50:length(y(:,2));%%%%Indicates the number of markers generated; the larger the middle value, the larger the marker interval

hold on

set(gca,'XTick',0:5:50,'YTick',0:0.1:1) %%[0:axis unit length:1]

axis([0 50 0 1])%%Range of horizontal and vertical axes

xlabel('time');%%Name of horizontal axis

ylabel('x');%%Name of vertical axis

set(gca,'XTick',0:5:50,'YTick',0:0.1:1) %%[0:axis unit length:1]

axis([0 50 0 1])%%Range of horizontal and vertical axes

xlabel('time');%%Name of horizontal axis

ylabel('y');%%Name of vertical axis

legend('\gamma=0', '\gamma=0.5', '\gamma=0.9','FontSize', 18); %%%Legend

% Set font properties for axes and labels

set(gca, 'FontName', 'Times New Roman', 'FontSize', 16);

xlabel('\it time', 'FontName', 'Times New Roman', 'FontSize', 16); % x-axis label

ylabel('\it y', 'FontName', 'Times New Roman', 'FontSize', 16); % y-axis label

% Add title below the subplot (b)

text(0.5, -0.12, '(b) {\ite_n}=5.2, {\ite_r}=0.52', ... % Lower y-coordinate position

'FontName', 'Times New Roman', 'FontSize', 16, ...

'HorizontalAlignment', 'center', ...

'VerticalAlignment', 'top', ...

'Units', 'normalized');

Fig.10

clear; clc; % Clear workspace, clear command line

% Create figure and adjust layout

figure;

% Adjust subplot position (leave 20% space at the bottom)

subplot('Position', [0.1, 0.15, 0.35, 0.8]); % [left, bottom, width, height]

% Phase portrait of the first system (en=0.1, er=0.075)

%%%%1

k=0.8;t=0.8;cn=0.5;cr=0.2;cq=0.2;g=0.4 ;a=0.5;er=0.52;en=0.7 ;derta=0.4;theta=0.5;f=0.8;gama=0;%Parameter assignment

[v,y]=ode45(@(v,y) liangfang_R_xiugai(v,y,k,t,cn,cr,cq,g,a,er,en,derta,theta,f,gama),[0,50],[0.5,0.5]); %Algorithm to solve the equation, equation running time (number of steps), initial values of x and y

p=plot(v,y(:,1),'ro--','linewidth',1.5,'markersize',8);

p.MarkerIndices = 1:15:length(y(:,1));%%%%Indicates the number of markers generated; the larger the middle value, the larger the marker interval

hold on

%%%%2

k=0.8;t=0.8;cn=0.5;cr=0.2;cq=0.2;g=0.4 ;a=0.5;er=0.52;en=0.7 ;derta=0.4;theta=0.5;f=0.8;gama=0.5;%Parameter assignment

[v,y]=ode45(@(v,y) liangfang_R_xiugai(v,y,k,t,cn,cr,cq,g,a,er,en,derta,theta,f,gama),[0,50],[0.5,0.5]); %Algorithm to solve the equation, equation running time (number of steps), initial values of x and y

p=plot(v,y(:,1),'m>--','linewidth',1.5,'markersize',8);

p.MarkerIndices = 1:15:length(y(:,1));%%%%Indicates the number of markers generated; the larger the middle value, the larger the marker interval

hold on

%%%%3

k=0.8;t=0.8;cn=0.5;cr=0.2;cq=0.2;g=0.4 ;a=0.5;er=0.52;en=0.7 ;derta=0.4;theta=0.5;f=0.8;gama=0.9;%Parameter assignment

[v,y]=ode45(@(v,y) liangfang_R_xiugai(v,y,k,t,cn,cr,cq,g,a,er,en,derta,theta,f,gama),[0,50],[0.5,0.5]); %Algorithm to solve the equation, equation running time (number of steps), initial values of x and y

p=plot(v,y(:,1),'bd--','linewidth',1.5,'markersize',8);

p.MarkerIndices = 1:15:length(y(:,1));%%%%Indicates the number of markers generated; the larger the middle value, the larger the marker interval

hold on

set(gca,'XTick',0:5:50,'YTick',0:0.1:1) %%[0:axis unit length:1]

axis([0 50 0 1])%%Range of horizontal and vertical axes

xlabel('time');%%Name of horizontal axis

ylabel('x');%%Name of vertical axis

legend('\delta=0.3', '\delta=0.4', '\delta=0.5','FontSize', 18); %%%Legend

% Set font properties for axes and labels

set(gca, 'FontName', 'Times New Roman', 'FontSize', 16);

xlabel('\it time', 'FontName', 'Times New Roman', 'FontSize', 16); % x-axis label

ylabel('\it x', 'FontName', 'Times New Roman', 'FontSize', 16); % y-axis label

% Add title below the subplot (a)

text(0.5, -0.12, '(a) {\ite_n}=0.1, {\ite_r}=0.075', ... % Lower y-coordinate position

'FontName', 'Times New Roman', 'FontSize', 16, ...

'HorizontalAlignment', 'center', ...

'VerticalAlignment', 'top', ...

'Units', 'normalized');

% Second subplot (adjust position)

subplot('Position', [0.55, 0.15, 0.35, 0.8]); % Symmetric to the first subplot

% Phase portrait of the second system (en=0.52, er=0.052)

%%%%1

k=0.8;t=0.8;cn=0.5;cr=0.2;cq=0.2;g=0.4 ;a=0.5;er=0.52;en=1.2 ;derta=0.3;theta=0.9;f=0.8;gama=0;%Parameter assignment

[v,y]=ode45(@(v,y) liangfang_R_xiugai(v,y,k,t,cn,cr,cq,g,a,er,en,derta,theta,f,gama),[0,50],[0.5,0.5]); %Algorithm to solve the equation, equation running time (number of steps), initial values of x and y

p=plot(v,y(:,1),'ro--','linewidth',1.5,'markersize',8);

p.MarkerIndices = 1:10:length(y(:,1));%%%%Indicates the number of markers generated; the larger the middle value, the larger the marker interval

hold on

%%%%2

k=0.8;t=0.8;cn=0.5;cr=0.2;cq=0.2;g=0.4 ;a=0.5;er=0.52;en=1.2 ;derta=0.4;theta=0.9;f=0.8;gama=0;%Parameter assignment

[v,y]=ode45(@(v,y) liangfang_R_xiugai(v,y,k,t,cn,cr,cq,g,a,er,en,derta,theta,f,gama),[0,50],[0.5,0.5]); %Algorithm to solve the equation, equation running time (number of steps), initial values of x and y

p=plot(v,y(:,1),'m>--','linewidth',1.5,'markersize',8);

p.MarkerIndices = 1:10:length(y(:,1));%%%%Indicates the number of markers generated; the larger the middle value, the larger the marker interval

hold on

%%%%3

k=0.8;t=0.8;cn=0.5;cr=0.2;cq=0.2;g=0.4 ;a=0.5;er=0.52;en=1.2 ;derta=0.5;theta=0.9;f=0.8;gama=0;%Parameter assignment

[v,y]=ode45(@(v,y) liangfang_R_xiugai(v,y,k,t,cn,cr,cq,g,a,er,en,derta,theta,f,gama),[0,50],[0.5,0.5]); %Algorithm to solve the equation, equation running time (number of steps), initial values of x and y

p=plot(v,y(:,1),'bd--','linewidth',1.5,'markersize',8);

p.MarkerIndices = 1:10:length(y(:,1));%%%%Indicates the number of markers generated; the larger the middle value, the larger the marker interval

hold on

set(gca,'XTick',0:5:50,'YTick',0:0.1:1) %%[0:axis unit length:1]

axis([0 50 0 1])%%Range of horizontal and vertical axes

xlabel('time');%%Name of horizontal axis

ylabel('x');%%Name of vertical axis

legend('\delta=0.3', '\delta=0.4', '\delta=0.5','FontSize', 18); %%%Legend

% Set font properties for axes and labels

set(gca, 'FontName', 'Times New Roman', 'FontSize', 16);

xlabel('\it time', 'FontName', 'Times New Roman', 'FontSize', 16); % x-axis label

ylabel('\it x', 'FontName', 'Times New Roman', 'FontSize', 16); % y-axis label

% Add title below the subplot (b)

text(0.5, -0.12, '(b) {\ite_n}=5.2, {\ite_r}=0.52', ... % Lower y-coordinate position

'FontName', 'Times New Roman', 'FontSize', 16, ...

'HorizontalAlignment', 'center', ...

'VerticalAlignment', 'top', ...

'Units', 'normalized');

Fig.11

clear; clc; % Clear workspace, clear command line

% Create figure and adjust layout

figure;

% Adjust subplot position (leave 20% space at the bottom)

subplot('Position', [0.1, 0.15, 0.35, 0.8]); % [left, bottom, width, height]

% Phase portrait of the first system (en=0.1, er=0.075)

%%%%1

k=0.8;t=0.8;cn=0.5;cr=0.2;cq=0.2;g=0.3 ;a=0.5;er=0.52;en=0.7 ;derta=0.2;theta=0.9;f=0.8;gama=0.9;%Parameter assignment

[v,y]=ode45(@(v,y) liangfang_R_xiugai(v,y,k,t,cn,cr,cq,g,a,er,en,derta,theta,f,gama),[0,50],[0.5,0.5]); %Algorithm to solve the equation, equation running time (number of steps), initial values of x and y

p=plot(v,y(:,2),'ro-','linewidth',1.5,'markersize',8);

p.MarkerIndices = 1:5:length(y(:,2));

%%Colors: y=yellow; k=black; w=white; b=blue; g=green; r=red; c=cyan; m=magenta

%%Marker styles: +, o, *, x, s=square, d=diamond, v=down triangle, >=right triangle, <=left triangle, p=pentagram, h=hexagram, none=no marker

%Line styles: -solid, --dashed, -.-dash-dot, .dotted

%linewidth = line width, markersize = marker size, MarkerIndices = marker interval, larger middle value in 1:1:length(t) means larger marker interval

hold on

%%%%2

k=0.8;t=0.8;cn=0.5;cr=0.2;cq=0.2;g=0.3 ;a=0.5;er=0.52;en=0.7 ;derta=0.4;theta=0.9;f=0.8;gama=0.9;%Parameter assignment

[v,y]=ode45(@(v,y) liangfang_R_xiugai(v,y,k,t,cn,cr,cq,g,a,er,en,derta,theta,f,gama),[0,50],[0.5,0.5]); %Algorithm to solve the equation, equation running time (number of steps), initial values of x and y

p=plot(v,y(:,2),'m>-','linewidth',1.5,'markersize',8);

p.MarkerIndices = 1:5:length(y(:,2));%%%%Indicates the number of markers generated; the larger the middle value, the larger the marker interval

hold on

%%%%3

k=0.8;t=0.8;cn=0.5;cr=0.2;cq=0.2;g=0.3 ;a=0.5;er=0.52;en=0.7 ;derta=0.6;theta=0.9;f=0.8;gama=0.9;%Parameter assignment

[v,y]=ode45(@(v,y) liangfang_R_xiugai(v,y,k,t,cn,cr,cq,g,a,er,en,derta,theta,f,gama),[0,50],[0.5,0.5]); %Algorithm to solve the equation, equation running time (number of steps), initial values of x and y

p=plot(v,y(:,2),'bd-','linewidth',1.5,'markersize',8);

p.MarkerIndices = 1:5:length(y(:,2));%%%%Indicates the number of markers generated; the larger the middle value, the larger the marker interval

hold on

set(gca,'XTick',0:5:50,'YTick',0:0.1:1) %%[0:axis unit length:1]

axis([0 50 0 1])%%Range of horizontal and vertical axes

xlabel('time');%%Name of horizontal axis

ylabel('x');%%Name of vertical axis

set(gca,'XTick',0:5:50,'YTick',0:0.1:1) %%[0:axis unit length:1]

axis([0 50 0 1])%%Range of horizontal and vertical axes

xlabel('time');%%Name of horizontal axis

ylabel('y');%%Name of vertical axis

legend('\delta=0.3', '\delta=0.4', '\delta=0.5','FontSize', 18); %%%Legend

% Set font properties for axes and labels

set(gca, 'FontName', 'Times New Roman', 'FontSize', 16);

xlabel('\it time', 'FontName', 'Times New Roman', 'FontSize', 16); % x-axis label

ylabel('\it y', 'FontName', 'Times New Roman', 'FontSize', 16); % y-axis label

% Add title below the subplot (a)

text(0.5, -0.12, '(a) {\ite_n}=0.1, {\ite_r}=0.075', ... % Lower y-coordinate position

'FontName', 'Times New Roman', 'FontSize', 16, ...

'HorizontalAlignment', 'center', ...

'VerticalAlignment', 'top', ...

'Units', 'normalized');

% Second subplot (adjust position)

subplot('Position', [0.55, 0.15, 0.35, 0.8]); % Symmetric to the first subplot

% Phase portrait of the second system (en=0.52, er=0.052)

%%%%1

k=0.8;t=0.8;cn=0.5;cr=0.2;cq=0.2;g=0.4 ;a=0.5;er=0.52;en=5.2;derta=0.2;theta=0.9;f=0.8;gama=0;%Parameter assignment

[v,y]=ode45(@(v,y) liangfang_R_xiugai(v,y,k,t,cn,cr,cq,g,a,er,en,derta,theta,f,gama),[0,50],[0.5,0.5]); %Algorithm to solve the equation, equation running time (number of steps), initial values of x and y

p=plot(v,y(:,2),'ro-','linewidth',1.5,'markersize',8);

p.MarkerIndices = 1:100:length(y(:,2));

%%Colors: y=yellow; k=black; w=white; b=blue; g=green; r=red; c=cyan; m=magenta

%%Marker styles: +, o, *, x, s=square, d=diamond, v=down triangle, >=right triangle, <=left triangle, p=pentagram, h=hexagram, none=no marker

%Line styles: -solid, --dashed, -.-dash-dot, .dotted

%linewidth = line width, markersize = marker size, MarkerIndices = marker interval, larger middle value in 1:1:length(t) means larger marker interval

hold on

%%%%2

k=0.8;t=0.8;cn=0.5;cr=0.2;cq=0.2;g=0.4 ;a=0.5;er=0.52;en=5.2 ;derta=0.3;theta=0.9;f=0.8;gama=0;%Parameter assignment

[v,y]=ode45(@(v,y) liangfang_R_xiugai(v,y,k,t,cn,cr,cq,g,a,er,en,derta,theta,f,gama),[0,50],[0.5,0.5]); %Algorithm to solve the equation, equation running time (number of steps), initial values of x and y

p=plot(v,y(:,2),'m>-','linewidth',1.5,'markersize',8);

p.MarkerIndices = 1:100:length(y(:,2));%%%%Indicates the number of markers generated; the larger the middle value, the larger the marker interval

hold on

%%%%3

k=0.8;t=0.8;cn=0.5;cr=0.2;cq=0.2;g=0.4 ;a=0.5;er=0.52;en=5.2 ;derta=0.4;theta=0.9;f=0.8;gama=0;%Parameter assignment

[v,y]=ode45(@(v,y) liangfang_R_xiugai(v,y,k,t,cn,cr,cq,g,a,er,en,derta,theta,f,gama),[0,50],[0.5,0.5]); %Algorithm to solve the equation, equation running time (number of steps), initial values of x and y

p=plot(v,y(:,2),'bd-','linewidth',1.5,'markersize',8);

p.MarkerIndices = 1:150:length(y(:,2));%%%%Indicates the number of markers generated; the larger the middle value, the larger the marker interval

hold on

set(gca,'XTick',0:5:50,'YTick',0:0.1:1) %%[0:axis unit length:1]

axis([0 50 0 1])%%Range of horizontal and vertical axes

xlabel('time');%%Name of horizontal axis

ylabel('x');%%Name of vertical axis

set(gca,'XTick',0:5:50,'YTick',0:0.1:1) %%[0:axis unit length:1]

axis([0 50 0 1])%%Range of horizontal and vertical axes

xlabel('time');%%Name of horizontal axis

ylabel('y');%%Name of vertical axis

legend('\delta=0.3', '\delta=0.4', '\delta=0.5','FontSize', 18); %%%Legend

% Set font properties for axes and labels

set(gca, 'FontName', 'Times New Roman', 'FontSize', 16);

xlabel('\it time', 'FontName', 'Times New Roman', 'FontSize', 16); % x-axis label

ylabel('\it y', 'FontName', 'Times New Roman', 'FontSize', 16); % y-axis label

% Add title below the subplot (b)

text(0.5, -0.12, '(b) {\ite_n}=5.2, {\ite_r}=0.52', ... % Lower y-coordinate position

'FontName', 'Times New Roman', 'FontSize', 16, ...

'HorizontalAlignment', 'center', ...

'VerticalAlignment', 'top', ...

'Units', 'normalized');

Fig.12

clear; clc; % Clear workspace, clear command line

% Create figure and adjust layout

figure;

% Adjust subplot position (leave 20% space at the bottom)

subplot('Position', [0.1, 0.15, 0.35, 0.8]); % [left, bottom, width, height]

% Phase portrait of the first system (en=0.1, er=0.075)

%%%%1

k=0.8;t=0.8;cn=0.5;cr=0.2;cq=0.2;g=0.4 ;a=0.5;er=0.52;en=0.7 ;derta=0.4;theta=0;f=0.8;gama=0.9;%Parameter assignment

[v,y]=ode45(@(v,y) liangfang_R_xiugai(v,y,k,t,cn,cr,cq,g,a,er,en,derta,theta,f,gama),[0,50],[0.5,0.5]); %Algorithm to solve the equation, equation running time (number of steps), initial values of x and y

p=plot(v,y(:,1),'ro--','linewidth',1.5,'markersize',8);

p.MarkerIndices = 1:15:length(y(:,1));%%%%Indicates the number of markers generated; the larger the middle value, the larger the marker interval

hold on

%%%%2

k=0.8;t=0.8;cn=0.5;cr=0.2;cq=0.2;g=0.4 ;a=0.5;er=0.52;en=0.7 ;derta=0.4;theta=0.5;f=0.8;gama=0.9;%Parameter assignment

[v,y]=ode45(@(v,y) liangfang_R_xiugai(v,y,k,t,cn,cr,cq,g,a,er,en,derta,theta,f,gama),[0,50],[0.5,0.5]); %Algorithm to solve the equation, equation running time (number of steps), initial values of x and y

p=plot(v,y(:,1),'m>--','linewidth',1.5,'markersize',8);

p.MarkerIndices = 1:15:length(y(:,1));%%%%Indicates the number of markers generated; the larger the middle value, the larger the marker interval

hold on

%%%%3

k=0.8;t=0.8;cn=0.5;cr=0.2;cq=0.2;g=0.4 ;a=0.5;er=0.52;en=0.7 ;derta=0.4;theta=0.9;f=0.8;gama=0.9;%Parameter assignment

[v,y]=ode45(@(v,y) liangfang_R_xiugai(v,y,k,t,cn,cr,cq,g,a,er,en,derta,theta,f,gama),[0,50],[0.5,0.5]); %Algorithm to solve the equation, equation running time (number of steps), initial values of x and y

p=plot(v,y(:,1),'bd--','linewidth',1.5,'markersize',8);

p.MarkerIndices = 1:15:length(y(:,1));%%%%Indicates the number of markers generated; the larger the middle value, the larger the marker interval

hold on

set(gca,'XTick',0:5:50,'YTick',0:0.1:1) %%[0:axis unit length:1]

axis([0 50 0 1])%%Range of horizontal and vertical axes

xlabel('time');%%Name of horizontal axis

ylabel('x');%%Name of vertical axis

legend('\theta=0', '\theta=0.5', '\theta=0.9','FontSize', 18); %%%Legend

% Set font properties for axes and labels

set(gca, 'FontName', 'Times New Roman', 'FontSize', 16);

xlabel('\it time', 'FontName', 'Times New Roman', 'FontSize', 16); % x-axis label

ylabel('\it x', 'FontName', 'Times New Roman', 'FontSize', 16); % y-axis label

% Add title below the subplot (a)

text(0.5, -0.12, '(a) {\ite_n}=0.1, {\ite_r}=0.075', ... % Lower y-coordinate position

'FontName', 'Times New Roman', 'FontSize', 16, ...

'HorizontalAlignment', 'center', ...

'VerticalAlignment', 'top', ...

'Units', 'normalized');

% Second subplot (adjust position)

subplot('Position', [0.55, 0.15, 0.35, 0.8]); % Symmetric to the first subplot

% Phase portrait of the second system (en=0.52, er=0.052)

%%%%1

k=0.8;t=0.8;cn=0.5;cr=0.2;cq=0.2;g=0.4 ;a=0.5;er=0.52;en=5.2 ;derta=0.4;theta=0;f=0.8;gama=0.9;%Parameter assignment

[v,y]=ode45(@(v,y) liangfang_R_xiugai(v,y,k,t,cn,cr,cq,g,a,er,en,derta,theta,f,gama),[0,50],[0.5,0.5]); %Algorithm to solve the equation, equation running time (number of steps), initial values of x and y

p=plot(v,y(:,1),'ro--','linewidth',1.5,'markersize',8);

p.MarkerIndices = 1:150:length(y(:,1));%%%%Indicates the number of markers generated; the larger the middle value, the larger the marker interval

hold on

%%%%2

k=0.8;t=0.8;cn=0.5;cr=0.2;cq=0.2;g=0.4 ;a=0.5;er=0.52;en=5.2 ;derta=0.4;theta=0.5;f=0.8;gama=0.9;%Parameter assignment

[v,y]=ode45(@(v,y) liangfang_R_xiugai(v,y,k,t,cn,cr,cq,g,a,er,en,derta,theta,f,gama),[0,50],[0.5,0.5]); %Algorithm to solve the equation, equation running time (number of steps), initial values of x and y

p=plot(v,y(:,1),'m>--','linewidth',1.5,'markersize',8);

p.MarkerIndices = 1:150:length(y(:,1));%%%%Indicates the number of markers generated; the larger the middle value, the larger the marker interval

hold on

%%%%3

k=0.8;t=0.8;cn=0.5;cr=0.2;cq=0.2;g=0.4 ;a=0.5;er=0.52;en=5.2 ;derta=0.4;theta=0.9;f=0.8;gama=0.9;%Parameter assignment

[v,y]=ode45(@(v,y) liangfang_R_xiugai(v,y,k,t,cn,cr,cq,g,a,er,en,derta,theta,f,gama),[0,50],[0.5,0.5]); %Algorithm to solve the equation, equation running time (number of steps), initial values of x and y

p=plot(v,y(:,1),'bd--','linewidth',1.5,'markersize',8);

p.MarkerIndices = 1:150:length(y(:,1));%%%%Indicates the number of markers generated; the larger the middle value, the larger the marker interval

hold on

set(gca,'XTick',0:5:50,'YTick',0:0.1:1) %%[0:axis unit length:1]

axis([0 50 0 1])%%Range of horizontal and vertical axes

xlabel('time');%%Name of horizontal axis

ylabel('x');%%Name of vertical axis

legend('\theta=0', '\theta=0.5', '\theta=0.9','FontSize', 18); %%%Legend

% Set font properties for axes and labels

set(gca, 'FontName', 'Times New Roman', 'FontSize', 16);

xlabel('\it time', 'FontName', 'Times New Roman', 'FontSize', 16); % x-axis label

ylabel('\it x', 'FontName', 'Times New Roman', 'FontSize', 16); % y-axis label

% Add title below the subplot (b)

text(0.5, -0.12, '(b) {\ite_n}=5.2, {\ite_r}=0.52', ... % Lower y-coordinate position

'FontName', 'Times New Roman', 'FontSize', 16, ...

'HorizontalAlignment', 'center', ...

'VerticalAlignment', 'top', ...

'Units', 'normalized');

% Control the position of the small graph % The following is the detail graph to be added, this position can be adjusted arbitrarily

axes('position',[0.6,0.25,0.1,0.2]);

%%%%1

k=0.8;t=0.8;cn=0.5;cr=0.2;cq=0.2;g=0.4 ;a=0.5;er=0.52;en=5.2 ;derta=0.4;theta=0;f=0.8;gama=0.9;%Parameter assignment

[v,y]=ode45(@(v,y) liangfang_R_xiugai(v,y,k,t,cn,cr,cq,g,a,er,en,derta,theta,f,gama),[0,50],[0.5,0.5]); %Algorithm to solve the equation, equation running time (number of steps), initial values of x and y

p=plot(v,y(:,1),'ro--','linewidth',1.5,'markersize',8);

p.MarkerIndices = 1:150:length(y(:,1));%%%%Indicates the number of markers generated; the larger the middle value, the larger the marker interval

hold on

%%%%2

k=0.8;t=0.8;cn=0.5;cr=0.2;cq=0.2;g=0.4 ;a=0.5;er=0.52;en=5.2 ;derta=0.4;theta=0.5;f=0.8;gama=0.9;%Parameter assignment

[v,y]=ode45(@(v,y) liangfang_R_xiugai(v,y,k,t,cn,cr,cq,g,a,er,en,derta,theta,f,gama),[0,50],[0.5,0.5]); %Algorithm to solve the equation, equation running time (number of steps), initial values of x and y

p=plot(v,y(:,1),'m>--','linewidth',1.5,'markersize',8);

p.MarkerIndices = 1:150:length(y(:,1));%%%%Indicates the number of markers generated; the larger the middle value, the larger the marker interval

hold on

%%%%3

k=0.8;t=0.8;cn=0.5;cr=0.2;cq=0.2;g=0.4 ;a=0.5;er=0.52;en=5.2 ;derta=0.4;theta=0.9;f=0.8;gama=0.9;%Parameter assignment

[v,y]=ode45(@(v,y) liangfang_R_xiugai(v,y,k,t,cn,cr,cq,g,a,er,en,derta,theta,f,gama),[0,50],[0.5,0.5]); %Algorithm to solve the equation, equation running time (number of steps), initial values of x and y

p=plot(v,y(:,1),'bd--','linewidth',1.5,'markersize',8);

p.MarkerIndices = 1:150:length(y(:,1));%%%%Indicates the number of markers generated; the larger the middle value, the larger the marker interval

hold on

axis([0 0.04 0.45 0.55])

Fig.13

clear; clc; % Clear workspace, clear command line

% Create figure and adjust layout

figure;

% Adjust subplot position (leave 20% space at the bottom)

subplot('Position', [0.1, 0.15, 0.35, 0.8]); % [left, bottom, width, height]

% Phase portrait of the first system (en=0.1, er=0.075)

%%%%1

k=0.8;t=0.8;cn=0.5;cr=0.2;cq=0.2;g=0.3 ;a=0.5;er=0.52;en=0.7 ;derta=0.2;theta=0;f=0.8;gama=0.9;%Parameter assignment

[v,y]=ode45(@(v,y) liangfang_R_xiugai(v,y,k,t,cn,cr,cq,g,a,er,en,derta,theta,f,gama),[0,50],[0.5,0.5]); %Algorithm to solve the equation, equation running time (number of steps), initial values of x and y

p=plot(v,y(:,2),'ro-','linewidth',1.5,'markersize',8);

p.MarkerIndices = 1:5:length(y(:,2));

%%Colors: y=yellow; k=black; w=white; b=blue; g=green; r=red; c=cyan; m=magenta

%%Marker styles: +, o, *, x, s=square, d=diamond, v=down triangle, >=right triangle, <=left triangle, p=pentagram, h=hexagram, none=no marker

%Line styles: -solid, --dashed, -.-dash-dot, .dotted

%linewidth = line width, markersize = marker size, MarkerIndices = marker interval, larger middle value in 1:1:length(t) means larger marker interval

hold on

%%%%2

k=0.8;t=0.8;cn=0.5;cr=0.2;cq=0.2;g=0.3 ;a=0.5;er=0.52;en=0.7 ;derta=0.2;theta=0.5;f=0.8;gama=0.9;%Parameter assignment

[v,y]=ode45(@(v,y) liangfang_R_xiugai(v,y,k,t,cn,cr,cq,g,a,er,en,derta,theta,f,gama),[0,50],[0.5,0.5]); %Algorithm to solve the equation, equation running time (number of steps), initial values of x and y

p=plot(v,y(:,2),'m>-','linewidth',1.5,'markersize',8);

p.MarkerIndices = 1:5:length(y(:,2));%%%%Indicates the number of markers generated; the larger the middle value, the larger the marker interval

hold on

%%%%3

k=0.8;t=0.8;cn=0.5;cr=0.2;cq=0.2;g=0.3 ;a=0.5;er=0.52;en=0.7 ;derta=0.2;theta=0.9;f=0.8;gama=0.9;%Parameter assignment

[v,y]=ode45(@(v,y) liangfang_R_xiugai(v,y,k,t,cn,cr,cq,g,a,er,en,derta,theta,f,gama),[0,50],[0.5,0.5]); %Algorithm to solve the equation, equation running time (number of steps), initial values of x and y

p=plot(v,y(:,2),'bd-','linewidth',1.5,'markersize',8);

p.MarkerIndices = 1:10:length(y(:,2));%%%%Indicates the number of markers generated; the larger the middle value, the larger the marker interval

hold on

set(gca,'XTick',0:5:50,'YTick',0:0.1:1) %%[0:axis unit length:1]

axis([0 50 0 1])%%Range of horizontal and vertical axes

xlabel('time');%%Name of horizontal axis

ylabel('x');%%Name of vertical axis

set(gca,'XTick',0:5:50,'YTick',0:0.1:1) %%[0:axis unit length:1]

axis([0 50 0 1])%%Range of horizontal and vertical axes

xlabel('time');%%Name of horizontal axis

ylabel('y');%%Name of vertical axis

legend('\theta=0', '\theta=0.5', '\theta=0.9','FontSize', 18); %%%Legend

% Set font properties for axes and labels

set(gca, 'FontName', 'Times New Roman', 'FontSize', 16);

xlabel('\it time', 'FontName', 'Times New Roman', 'FontSize', 16); % x-axis label

ylabel('\it y', 'FontName', 'Times New Roman', 'FontSize', 16); % y-axis label

% Add title below the subplot (a)

text(0.5, -0.12, '(a) {\ite_n}=0.1, {\ite_r}=0.075', ... % Lower y-coordinate position

'FontName', 'Times New Roman', 'FontSize', 16, ...

'HorizontalAlignment', 'center', ...

'VerticalAlignment', 'top', ...

'Units', 'normalized');

% Second subplot (adjust position)

subplot('Position', [0.55, 0.15, 0.35, 0.8]); % Symmetric to the first subplot

% Phase portrait of the second system (en=0.52, er=0.052)

%%%%1

k=0.8;t=0.8;cn=0.5;cr=0.2;cq=0.2;g=0.3 ;a=0.5;er=0.52;en=5.2 ;derta=0.2;theta=0;f=0.8;gama=0.9;%Parameter assignment

[v,y]=ode45(@(v,y) liangfang_R_xiugai(v,y,k,t,cn,cr,cq,g,a,er,en,derta,theta,f,gama),[0,50],[0.5,0.5]); %Algorithm to solve the equation, equation running time (number of steps), initial values of x and y

p=plot(v,y(:,2),'ro-','linewidth',1.5,'markersize',8);

p.MarkerIndices = 1:50:length(y(:,2));

%%Colors: y=yellow; k=black; w=white; b=blue; g=green; r=red; c=cyan; m=magenta

%%Marker styles: +, o, *, x, s=square, d=diamond, v=down triangle, >=right triangle, <=left triangle, p=pentagram, h=hexagram, none=no marker

%Line styles: -solid, --dashed, -.-dash-dot, .dotted

%linewidth = line width, markersize = marker size, MarkerIndices = marker interval, larger middle value in 1:1:length(t) means larger marker interval

hold on

%%%%2

k=0.8;t=0.8;cn=0.5;cr=0.2;cq=0.2;g=0.3 ;a=0.5;er=0.52;en=5.2 ;derta=0.2;theta=0.5;f=0.8;gama=0.9;%Parameter assignment

[v,y]=ode45(@(v,y) liangfang_R_xiugai(v,y,k,t,cn,cr,cq,g,a,er,en,derta,theta,f,gama),[0,50],[0.5,0.5]); %Algorithm to solve the equation, equation running time (number of steps), initial values of x and y

p=plot(v,y(:,2),'m>-','linewidth',1.5,'markersize',8);

p.MarkerIndices = 1:50:length(y(:,2));%%%%Indicates the number of markers generated; the larger the middle value, the larger the marker interval

hold on

%%%%3

k=0.8;t=0.8;cn=0.5;cr=0.2;cq=0.2;g=0.3 ;a=0.5;er=0.52;en=5.2 ;derta=0.2;theta=0.9;f=0.8;gama=0.9;%Parameter assignment

[v,y]=ode45(@(v,y) liangfang_R_xiugai(v,y,k,t,cn,cr,cq,g,a,er,en,derta,theta,f,gama),[0,50],[0.5,0.5]); %Algorithm to solve the equation, equation running time (number of steps), initial values of x and y

p=plot(v,y(:,2),'bd-','linewidth',1.5,'markersize',8);

p.MarkerIndices = 1:150:length(y(:,2));%%%%Indicates the number of markers generated; the larger the middle value, the larger the marker interval

hold on

set(gca,'XTick',0:5:50,'YTick',0:0.1:1) %%[0:axis unit length:1]

axis([0 50 0 1])%%Range of horizontal and vertical axes

xlabel('time');%%Name of horizontal axis

ylabel('x');%%Name of vertical axis

set(gca,'XTick',0:5:50,'YTick',0:0.1:1) %%[0:axis unit length:1]

axis([0 50 0 1])%%Range of horizontal and vertical axes

xlabel('time');%%Name of horizontal axis

ylabel('y');%%Name of vertical axis

legend('\theta=0', '\theta=0.5', '\theta=0.9','FontSize', 18); %%%Legend

% Set font properties for axes and labels

set(gca, 'FontName', 'Times New Roman', 'FontSize', 16);

xlabel('\it time', 'FontName', 'Times New Roman', 'FontSize', 16); % x-axis label

ylabel('\it y', 'FontName', 'Times New Roman', 'FontSize', 16); % y-axis label

% Add title below the subplot (b)

text(0.5, -0.12, '(b) {\ite_n}=5.2, {\ite_r}=0.52', ... % Lower y-coordinate position

'FontName', 'Times New Roman', 'FontSize', 16, ...

'HorizontalAlignment', 'center', ...

'VerticalAlignment', 'top', ...

'Units', 'normalized');
